# Spatial considerations in the resolution of inflammation: Elucidating leukocyte interactions via an experimentally-calibrated agent-based model

**DOI:** 10.1371/journal.pcbi.1008413

**Published:** 2020-11-02

**Authors:** Anahita Bayani, Joanne L. Dunster, Jonathan J. Crofts, Martin R. Nelson

**Affiliations:** 1 Department of Physics & Mathematics, Nottingham Trent University, Clifton Campus, Nottingham, NG11 8NS, United Kingdom; 2 Institute for Cardiovascular and Metabolic Research, University of Reading, Reading, RG6 6AS, United Kingdom; University of Pittsburgh, UNITED STATES

## Abstract

Many common medical conditions (such as cancer, arthritis, chronic obstructive pulmonary disease (COPD), and others) are associated with inflammation, and even more so when combined with the effects of ageing and multimorbidity. While the inflammatory response varies in different tissue types, under disease and in response to therapeutic interventions, it has common interactions that occur between immune cells and inflammatory mediators. Understanding these underlying inflammatory mechanisms is key in progressing treatments and therapies for numerous inflammatory conditions. It is now considered that constituent mechanisms of the inflammatory response can be actively manipulated in order to drive resolution of inflammatory damage; particularly, those mechanisms related to the pro-inflammatory role of neutrophils and the anti-inflammatory role of macrophages. In this article, we describe the assembly of a hybrid mathematical model in which the spatial spread of inflammatory mediators is described through partial differential equations, and immune cells (neutrophils and macrophages) are described individually via an agent-based modelling approach. We pay close attention to how immune cells chemotax toward pro-inflammatory mediators, presenting a model for cell chemotaxis that is calibrated against experimentally observed cell trajectories in healthy and COPD-affected scenarios. We illustrate how variations in key model parameters can drive the switch from resolution of inflammation to chronic outcomes, and show that aberrant neutrophil chemotaxis can move an otherwise healthy outcome to one of chronicity. Finally, we reflect on our results in the context of the on-going hunt for new therapeutic interventions.

## Author summary

Inflammation is the body’s primary defence to harmful stimuli such as infections, toxins and tissue strain but also underlies a much broader range of conditions, including asthma, arthritis and cancer. The inflammatory response is key in resolving injury to facilitate recovery, and involves a range of interactions between immune cells (leukocytes, neutrophils and macrophages in particular) and inflammatory mediators. Immune cells are recruited from the blood stream in response to injury. Once in tissue, neutrophils release toxins to kill invading agents and resolve damage; however, if not carefully managed by other immune cells (mainly macrophages), their responses can increase inflammation instead of helping to resolve it. We model these interactions in response to damage using a spatial model, examining how a healthy response can prevent localised inflammation from spreading. We pay close attention to how cells migrate toward the damaged area, as many inflammatory conditions are associated with impairment of this process. We calibrate our model against experimentally-observed cell trajectories from healthy patients and patients with chronic obstructive pulmonary disease. We illustrate that a healthy outcome depends strongly upon efficient cell migration and a delicate balance between the pro- and anti-inflammatory effects of neutrophils and macrophages.

## Introduction

A wealth of medical conditions, including cancer, arthritis, and chronic obstructive pulmonary disease (COPD), to name just a few, are underpinned by inflammation of the affected tissue. The acute inflammatory response is a broad term encompassing interactions between numerous key immune cells, which act to mitigate against inflammatory damage. The efficacy of this response acts as a key switch between restoring the healthy state or, if impaired, leading to chronic inflammatory damage. Understanding the acute inflammatory response is therefore key to progressing our understanding of these myriad conditions. Furthermore, the long-held view that the acute inflammatory response is a passive process has, in recent years, become superseded by school of thought that the active manipulation of its constituent mechanisms exhibits significant promise in the hunt for new therapies [1]. In this work, we aim to provide insight into the interactions between key immune cells and inflammatory mediators via a mathematical model of generic inflammation that offers scope to be tailored to specific ailments, with a view to informing future therapeutic targets. In particular, we aim to elucidate the extent to which localised tissue damage can spread spatially to invade neighbouring healthy tissue, paying particularly close attention to the role efficient neutrophil chemotaxis plays in resolving inflammation.

While the range of inflammation-related pathologies is broad, the cellular and chemical interactions that comprise the inflammatory response are largely similar across ailments [2]. The initial response primarily involves recruitment of leukocytes (neutrophils and macrophages) from the vasculature into the affected tissue. Neutrophils are recruited early and release substances that can kill bacteria, but which can also be detrimental to healthy tissue. The lifespan of neutrophils is short (typically from hours to days [3, 4]), and the apoptosis of neutrophils presents significant risk to the tissue, since their eventual necrosis results in the release of the cells’ toxic contents, causing more damage to the tissue [5]. This risk is mitigated by macrophages, which are recruited later and phagocytose foreign particles and dead or dying cells, including apoptotic neutrophils; the phagocytosis of apoptotic neutrophils thus minimises further tissue damage [5, 6]. The recruitment of these immune cells and the interactions between them are regulated by a variety of both pro- and anti-inflammatory mediators. The recruitment of neutrophils essentially provides a net positive feedback loop that can cause further tissue damage via release of pro-inflammatory mediators, while the recruitment of macrophages provides two negative feedback loops comprising the release of anti-inflammatory mediators (thus reducing neutrophil recruitment) and the phagocytosis of apoptotic neutrophils.

Central to the question of whether the above cellular interactions yield resolution of damage is the question of how efficiently relevant leukocytes are recruited to the damaged tissue. Numerous inflammatory conditions are associated with aberrant migration of neutrophils, in particular, while detrimental changes in neutrophil migration have also been observed in ageing, with neutrophils generally exhibiting reduced chemotaxis. (See *e.g.* [7–9] and references therein.) The *in-vitro* study of [10] used time-lapse photography to record trajectories of healthy and COPD-affected neutrophils migrating up gradients of interleukin-8 (IL-8), demonstrating that healthy neutrophils chemotax more efficiently, while impaired neutrophils can have weaker sensitivity to the local chemoattractant gradient, which we associate with an impaired ability to resolve tissue damage. We use the quantitative measurements of directed neutrophil motility given by [10] to calibrate our description of leukocyte chemotaxis in the model presented below.

Several authors have previously presented mathematical models of the inflammatory response, most commonly using ordinary or partial differential equations (ODEs, PDEs) to describe simplified groups of constituent cells and mediators. The early work of Lauffenburger and colleagues highlighted how the effectiveness of the inflammatory response depends critically upon the rates of diffusion and chemotaxis of inflammatory cells [11, 12]. Taking a spatially-averaged approach, Kumar *et al.* [13] presented a model of a generic pathogen that included an early immune response and a late pro-inflammatory feedback, and suggested various therapies for persistent inflammation with the late feedback being a particular target for manipulation. Reynolds *et al.* [14] built upon this work to illustrate how modulation of a time-dependent anti-inflammatory response could also present a route to therapeutic interventions. Focusing upon understanding spatial interactions between generic groups of inflammatory cells, chemokines and anti-inflammatory cytokines, Penner *et al.* [15] used a reaction–diffusion (PDE) model to demonstrate how variations in key parameters can give rise to spatial patterns such as travelling waves, localised breathers and spatially inhomogeneous oscillations. The work of Dunster *et al.* [16] used a dynamical systems approach to analyse a homogeneous (ODE) model that included a thorough catalogue of interactions between pro- and anti-inflammatory mediators, macrophages and active and apoptotic neutrophils, identifying the rates of neutrophil apoptosis and macrophage phagocytosis as key targets for future therapies. This work was expanded to a spatial setting by Bayani *et al.* [17], who showed that spatially inhomogeneous configurations are permissible close to the switch from chronicity to health predicted by the original ODE model, in a manner that is sensitively controlled by the rates of mediator diffusion and immune cell diffusion and chemotaxis.

In all of the above models, the differential-equation-based modelling paradigm presents some limitations in terms of the ease of incorporating a full repertoire of relevant cells and interactions, which many authors circumvent by focusing on just the most key mechanisms or grouping together key components. Furthermore, differential equation models do not reflect the individual behaviours of each cell, and do not capture the inherent stochasticity in the response. An alternative approach is to use ‘cellular automata’ or ‘agent-based’ models (ABM), in which the behaviours of individual cells are described by a set of (usually stochastic) update rules. This modelling strategy has been utilised successfully in a range of biological applications, including tumour growth [18], bone remodelling [19], immune responses in the gut [20] and sexually-transmitted infections [21]. Within the context of generic inflammation, an ABM approach has been deployed by authors including Liepe *et al.* [22], who used approximate Bayesian computation to calibrate models of leukocyte dynamics against experimental data, and An, Vodovotz and coworkers, who have applied agent-based models to applications including diabetic foot ulcers, vocal fold injury and wound healing. (See [23, 24] and references therein.) Cockrell and An [25] used an agent-based model of systemic inflammation to examine the controllability of sepsis via genetic algorithms that can push persistent non-recovering inflammation to a state of health. With the longer-term goal of incorporating more advanced machine learning algorithms, the authors identified the scope to use their work as a foundation for future studies that could progress the goal of personalised medicine, in which intervention strategies are bespoke to the patient’s individual condition. Existing literature also includes examples in which hybrid approaches are utilised, in which ABM are integrated with continuum descriptions such as differential equations. These hybrid models offer a wealth of advantages over both ODE/PDE-based models and classical ABM, since they combine the cell-scale detail and stochasticity offered by ABM with the computational convenience of using differential equations to model aspects that are well approximated by continuum descriptions. Solovyev *et al.* [26] used a hybrid modelling approach to construct a model of pressure ulcer formation in patients with spinal chord injury, in which contributory mechanisms related to blood flow were modelled via ODEs and skin injury, inflammation and ulcer formation were modelled via ABM. Their model predicts that patients with spinal chord injury are at greater risk of developing pressure ulcers. Furthermore, the model of Bhui and Hayenga [27] used a hybrid approach to model leukocyte migration during atherogenesis, in which cellular behaviours were modelled via ABM and haemodynamic effects were modelled via computational fluid dynamics. The authors’ multiscale approach was successful in replicating leukocyte migration dynamics and the formation of atherosclerotic plaques.

In this paper, we describe the assembly of a hybrid PDE–ABM of the acute inflammatory response, in which generic groups of pro- and anti-inflammatory mediators are modelled via PDEs (in a manner similar to that of [17]) and groups of key immune cells (macrophages and both active and apoptotic neutrophils) are incorporated through an agent-based approach. In doing so, our principal aim is to provide new insight into spatial aspects of spreading inflammation, addressing the questions of how localised tissue damage can invade neighbouring healthy tissue, and how the spatial motility of constituent components of the inflammatory response can be manipulated to promote restoration of health. While previous models often restrict attention to specific tissues or inflammatory conditions, we present a generic model that has relevance spanning multiple scenarios. Given the significance of chemotaxis in previous works, we pay particular attention to how to describe accurately the chemotactic motion of cells, and calibrate our resulting chemotaxis model against the experimental data of [10], for healthy patients and patients with COPD. Our goal here is not to provide new insight in to COPD *per se*; it is to illustrate the manner in which our model can be easily tuned to relevant cell motility data in order to accurately describe specific conditions of interest in the future. We then proceed to elucidate the dependence of the inflammatory outcome upon each individual cellular behaviour, and examine the model’s sensitivity to related parameters. We draw brief qualitative comparison with the PDE model of [17], before finally reflecting on the results of our simulations within the broad context of the continuing search for inflammatory interventions.

## Methods

We, here, explain the construction and parameterisation of our model, beginning with preliminaries related to choices of domain and initial conditions, before describing the constitutive PDEs used in modelling the spatial distribution and spread of inflammatory mediators and the stochastic rules that govern cell behaviour. We then focus in particular upon the manner in which we model chemotactic cell motion in response to inflammatory damage. We implement our model via the existing agent-based modelling platform Repast Simphony (which is described in detail in [28]); the source code used to produce our simulations is available via github. Briefly, our code consists of the interactions between four classes: an Environment class, which contains the implementation of the PDEs that govern pro- and anti-inflammatory mediators, and the classes Macrophage, Neutrophil and Apoptotic, which describe agents representing macrophages, active neutrophils and apoptotic neutrophils respectively. Repast Simphony offers the advantage that agents can easily be provided with scheduled methods that act to evolve the agent at each ‘tick’ of the simulation. The interactions between the above classes are managed by an over-arching Builder class, which is also responsible for configuring the spatial domain, together with initial and boundary conditions, as described below.

### Domain and initial conditions

**Fig 1:**
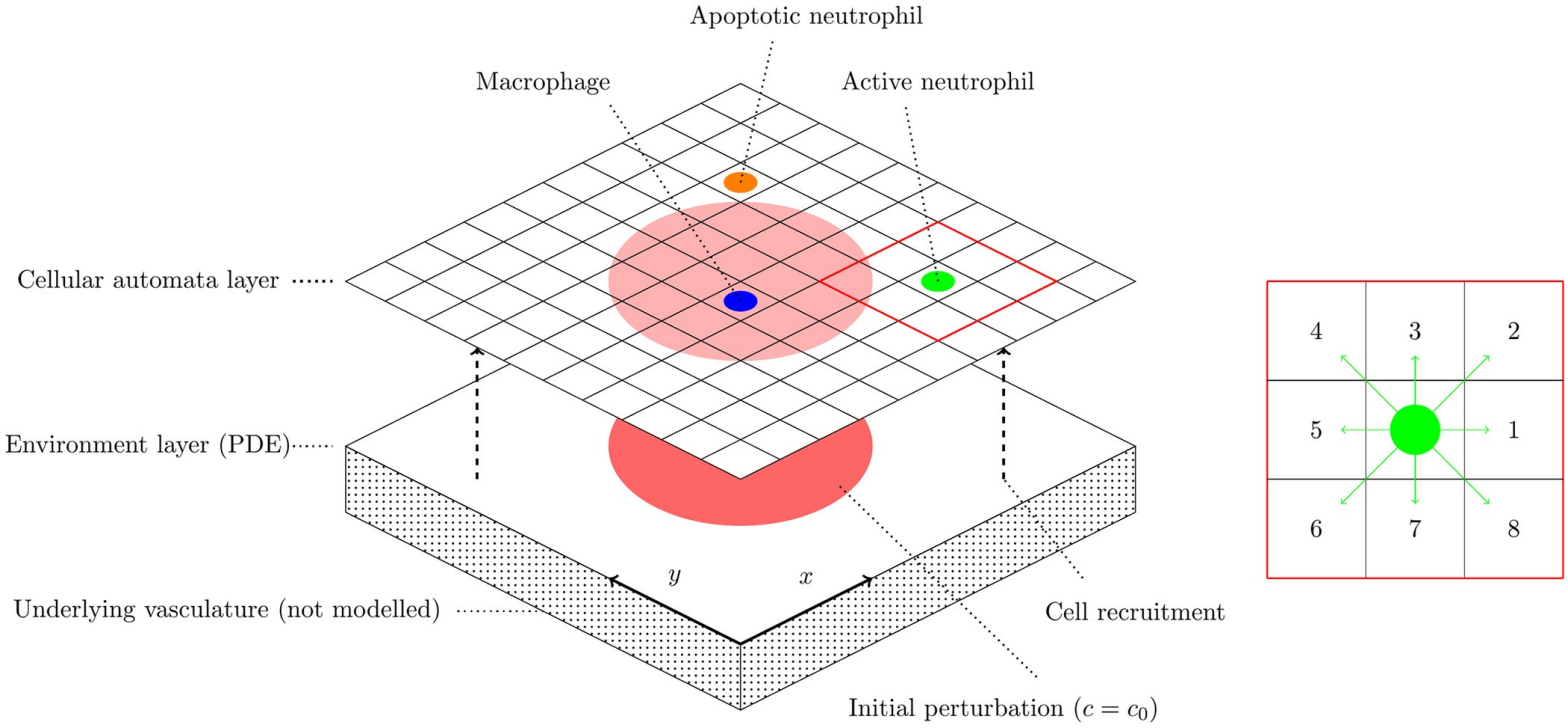
Schema of the spatial domain used in our simulations. Inset: the Moore neighbourhood of a generic cell, showing the indexing system used to number neighbouring grid spaces when defining and calibrating our model of cell chemotaxis.

In our simulations, agents are placed within a square/rectangular region that represents the model’s spatial domain, as illustrated in Fig 1. With the exception of when considering a reduced model via which we calibrate our description of chemotaxis below, in which we choose our domain to match corresponding experiments, we configure our domain and initial conditions as follows. We simulate our model on a 100 × 100 spatial grid. The domain is subject to periodic boundary conditions on all sides.

We note that the processes that underlie tissue damage are broad and can vary between tissues and ailments. Since our model is generic and constructed to have applicability spanning multiple inflammatory scenarios, we do not incorporate a specific description of tissue damage in our model. Instead, we treat the concentration of pro-inflammatory mediators present at any spatial location as a convenient proxy for tissue damage, and regard an absence of pro-inflammatory components (mediators and neutrophils) as a marker for restoration of health. We note that this approach comprises a degree of simplification for tractability; for example, it is conceivable that there may exist configurations in which pro-inflammatory mediator concentrations are instantaneously high, while inflammation is low due to *e.g.* high-concentrations of anti-inflammatory mediators. This notwithstanding, long-term inflammatory outcomes do correlate with concentrations of pro-inflammatory mediators, with some pro-inflammatory mediators (such as interleukin-6) commonly being used as biomarkers for progression of inflammatory conditions [29].

**Fig 2:**
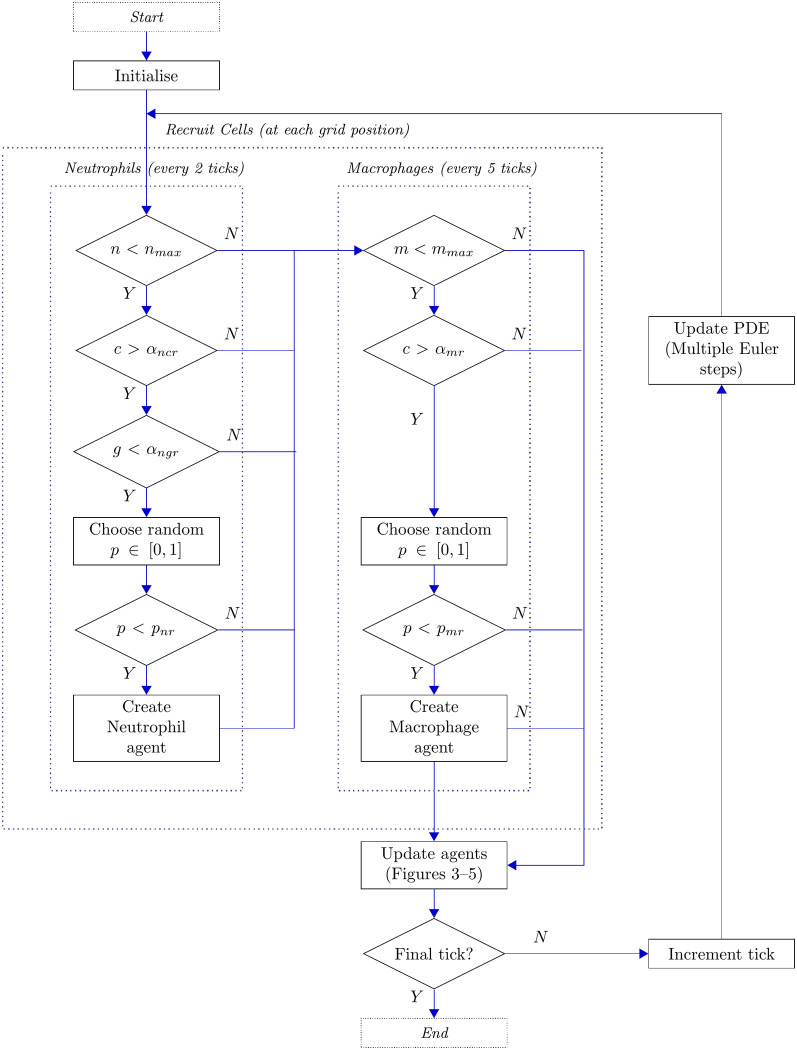
Flow chart illustrating the overarching cell recruitment and update structure of the model. Here, *m* and *n* represent the total number of macrophages and neutrophils in the domain respectively.

Owing to the above, our simulations focus on the question of how the cascade of inflammatory responses act to resolve perturbations to the healthy state. Specifically, we perturb the healthy state by setting the pro-inflammatory mediator concentration to some positive value, *c* = *c*_0_, within a circular area of radius *r* in the centre of the domain. (This perturbation acts as our proxy for initial tissue damage.) Outside of this area, *c* = 0. We initialise our simulations with no cells and no anti-inflammatory mediators. This initial concentration of pro-inflammatory mediators then drives recruitment of cells according to the flow chart illustrated in Fig 2, and as described in the following sections. The interaction and evolution of individual agents will be elucidated in subsequent sections and figures.

### Modelling mediators

We model the spatial distribution and spread of pro- and anti-inflammatory mediators (denoted *c* and *g* respectively) via reaction–diffusion PDEs. We denote by *D*_*c*_ and *D*_*g*_ the diffusion coefficients of each mediator, and introduce associated decay rates *γ*_*c*_ and *γ*_*g*_ respectively. The evolution of the mediators is then governed by the equations

$$\begin{array}{cc} \frac{\partial c}{\partial t} & =D_{c}\Delta^{2}c-\gamma_{c}c+\Gamma_{c}, \end{array}$$ (1)

$$\begin{array}{cc} \frac{\partial g}{\partial t} & =D_{g}\Delta^{2}g-\gamma_{g}g+\Gamma_{g}. \end{array}$$ (2)

In the above, Γ_*c*_ and Γ_*g*_ incorporate any sources of mediators owing to the actions of the cellular agents described below. Eqs (1) and (2) are solved numerically on the discrete grid described above; the numerical scheme employs a five-point Laplacian for the diffusive term and utilises Euler’s method for the timestepping, with an associated timestep d*t*. Note that we tune d*t* to ensure numerical stability, and in doing so take multiple numerical timesteps per tick of the ABM algorithm.

### Macrophages

**Fig 3:**
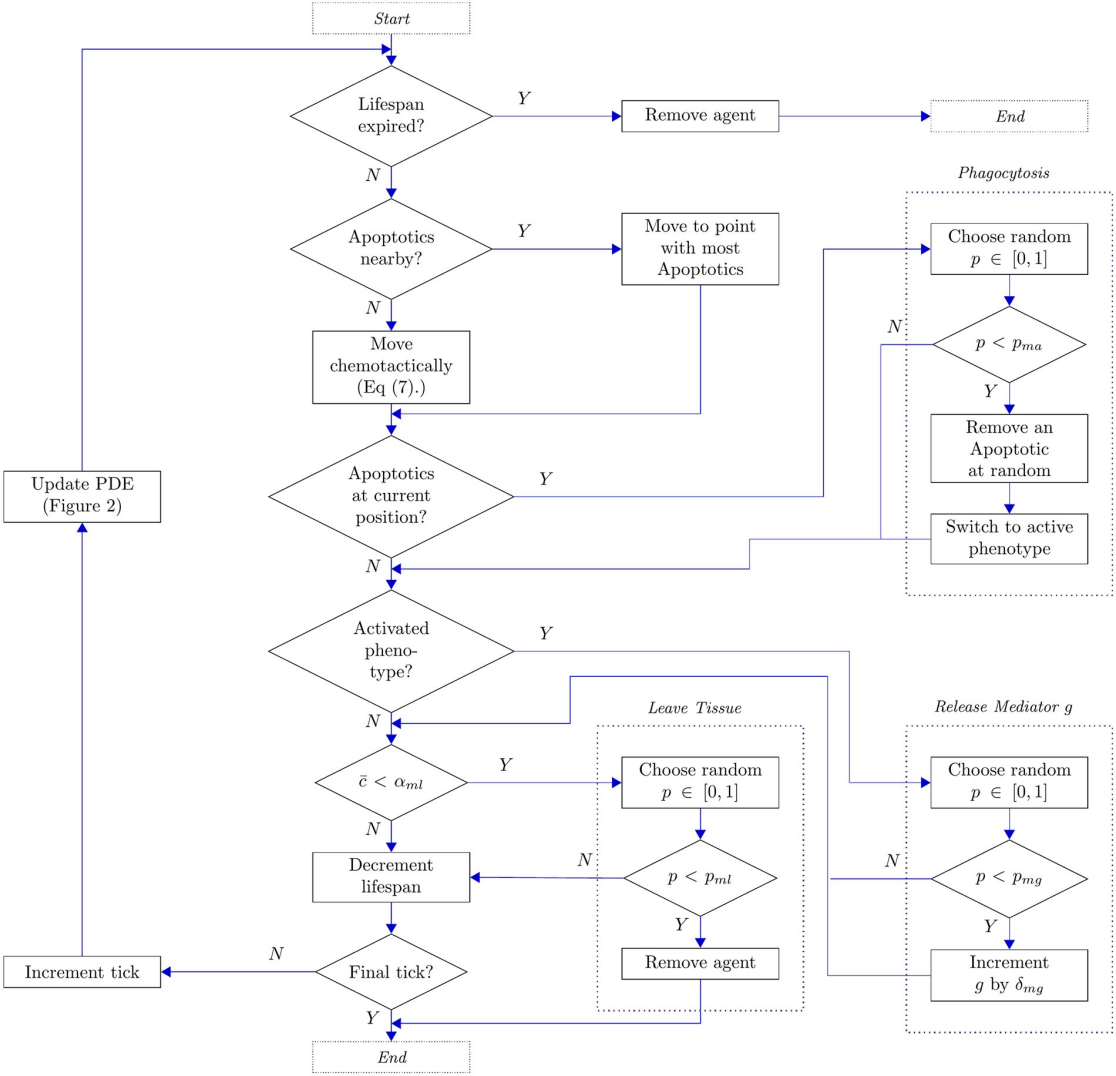
Flow chart illustrating the actions of each agent of the macrophage class. Here, $\bar{c}$ represents the total concentration of mediator *c* in the neighbourhood of the agent.

Macrophages are recruited to damaged tissue in order to regulate inflammatory responses and mitigate against further damage by apoptotic neutrophils. In our model, at each tick, each macrophage agent carries out four actions: it decides whether to move (and, if so, where to); it attempts to remove apoptotic neutrophils in its neighbourhood; it potentially releases anti-inflammatory mediators and it decides whether to vacate the tissue (if inflammation is sufficiently resolved). These hierarchical actions are illustrated in the flow chart shown in Fig 3; we expand upon how these behaviours are implemented below.

#### Macrophage recruitment

In the presence of suitably high concentrations of pro-inflammatory mediators, macrophages are recruited to the damaged tissue in order to counteract the inflammation. In order to model the natural delay between inflammatory damage and mediator recruitment, we schedule recruitment of macrophages every five ticks. Macrophages are recruited subject to a probability *p*_*mr*_ provided that the local pro-inflammatory mediator concentration is above a threshold *α*_*mr*_; this is, at each grid point, if *c* > *α*_*mr*_, macrophage agents are recruited with probability *p*_*mr*_. To avoid the biologically unrealistic possibility of infinite recruitment and accumulation of cells, we prescribe a maximum global number of macrophages *m*_*max*_ to be recruited within the tissue. We will infer this saturation level from available experimental data, as explained in the parameterisation section below. Upon recruitment, macrophages are configured with a lifespan, drawn from an appropriate distribution, which we also discuss below. This lifespan represents the duration for which the macrophage is expected to be in a biologically active state. This is broadly of the order of days and in general is observed to be much longer than that of neutrophils [30, 31]. Typically, macrophages live longer than a typical simulation presented here; however, if this lifespan is exceeded, the cell is removed from the simulation.

#### Macrophage motion

Macrophages are known to actively migrate toward apoptotic neutrophils, with the aim of phagocytosing these before they lyse and cause further inflammatory damage [32]. We thus configure our macrophage agents to move preferentially towards a neighbouring location that contains apoptotic neutrophils; if multiple neighbouring sites contain apoptotic cells, then one of these is selected at random. In the absence of any apoptotic neutrophils in any given macrophage’s neighbourhood, its preferential action is to undergo chemotaxis towards higher concentrations of pro-inflammatory mediators. We discuss the manner in which this chemotaxis is implemented in a dedicated section below. In the absence of both apoptotic neutrophils and pro-inflammatory mediators in the agent’s neighbourhood, the macrophage moves to a random neighbouring location.

#### Phagocytosis of apoptotic neutrophils

Macrophages act to clear apoptotic neutrophils through phagocytosis, and in doing so can promote the anti-inflammatory response. In our model, the phagocytosis of apoptotic neutrophils by macrophages is scheduled at every tick and simply consists, for each individual macrophage, of randomly selecting an apoptotic cell at its current position, if there are any, and removing it with probability *p*_*ma*_.

#### Release of anti-inflammatory mediators

It is well known that macrophages can exhibit a diverse range of behaviours, the selection of which relates to phenotypic switching that occurs upon environmental stimuli [33, 34]. Macrophages are commonly grouped into two broad subtypes: the classically activated M1 subtype, and the alternatively activated M2 subtype. Anti-inflammatory responses are associated with the latter of these [34]. We make the simple modelling assumption that the phenotypic switch that activates the anti-inflammatory response occurs when a given macrophase first phagocytoses an apoptotic neutrophil. Prior to this, that macrophage does not release any mediators. Following its first phagocytic activity, the macrophage yields an anti-inflammatory response in which (at every tick) the concentration of anti-inflammatory mediator at the macrophage’s location is increased by a quantity *δ*_*mg*_ with probability *p*_*mg*_.

#### Macrophage departure

Upon resolution of damage, we allow macrophages to vacate the modelled tissue, as observed in previous studies [35–37]. In order to do so, the total concentration of pro-inflammatory mediators in a given macrophage’s neighbourhood is assessed and if this is below a threshold *α*_*ml*_ that macrophage then leaves the tissue with probability *p*_*ml*_. The corresponding agent is then immediately removed from the simulation.

### Active neutrophils

**Fig 4:**
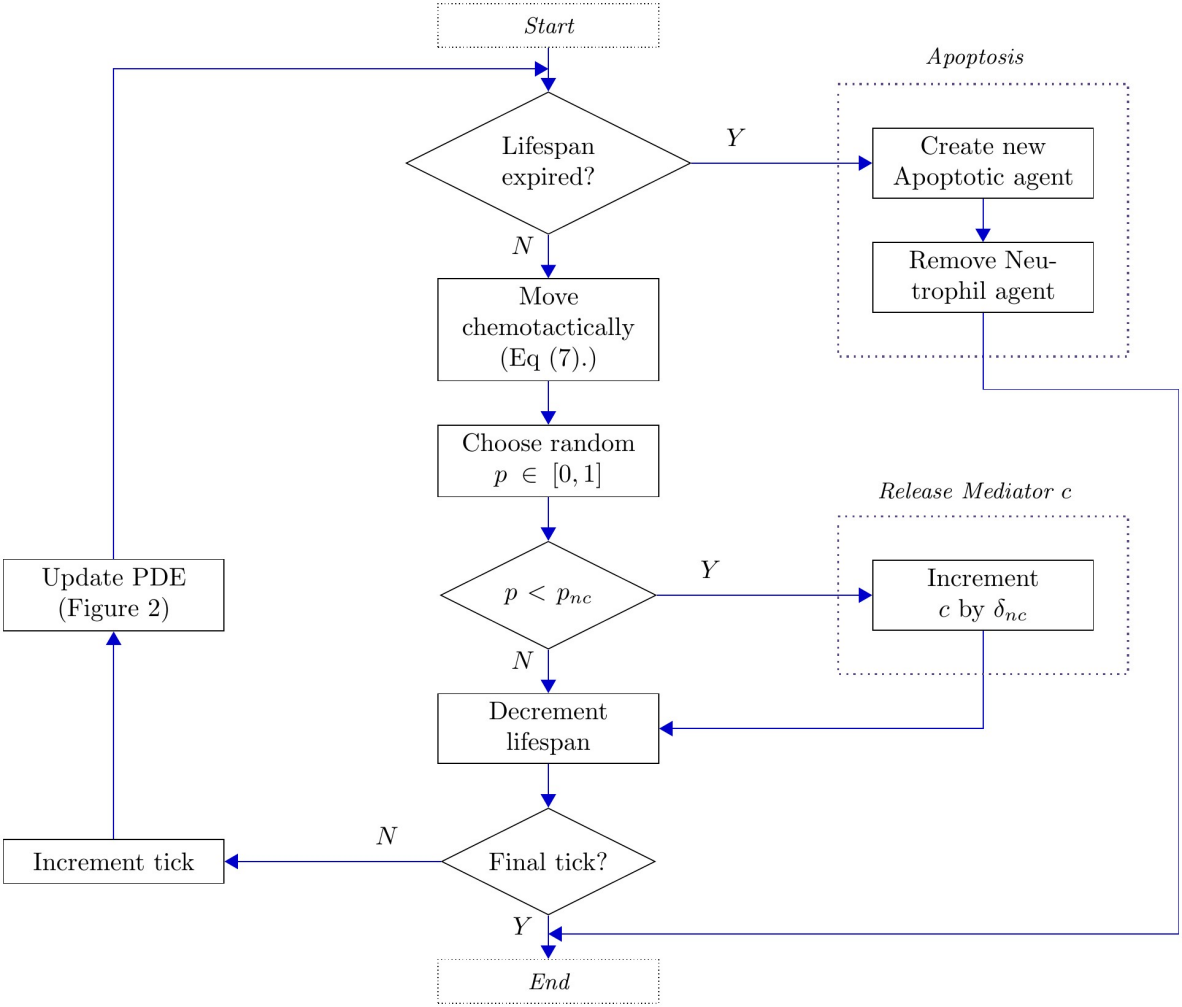
Flow chart illustrating the actions of each neutrophil agent.

Neutrophils are recruited to areas of tissue damage in response to high levels of pro-inflammatory mediators, but in a manner that depends also upon local concentrations of anti-inflammatory mediators [38]. Since neutrophils are recruited more rapidly than macrophages in general [39], we schedule the recruitment of neutrophils every two ticks. Neutrophils themselves can further contribute to the damage by continuous release of pro-inflammatory mediators. Neutrophils are relatively short-lived cells, and upon their eventual apoptosis can release large amounts of their toxic cell contents, further enhancing inflammation. A schematic representation of the set of rules regulating each neutrophil’s activity is provided in Fig 4; we expand upon the implementation of these behaviours below.

#### Neutrophil recruitment

Unlike the recruitment of macrophages described above, we consider neutrophil recruitment to be dependent upon the concentrations of both pro- and anti-inflammatory mediators; when the pro-inflammatory mediator concentration, *c*, is high and the anti-inflammatory mediator concentration, *g*, is low, we recruit neutrophils with a given probability. In our model, if both *c* > *α*_*ncr*_ and *g* < *α*_*ngr*_ we recruit a new neutrophil with probability *p*_*nr*_. As with macrophages, we prescribe a maximum global number of neutrophils in the system, *n*_*max*_, to avoid infinite recruitment and accumulation of cells within the tissue. Further details are provided in the dedicated parametrisation below. Once again, when a new neutrophil is added to the system, it is assigned a lifespan drawn from an appropriate distribution. In this case, this lifespan represents the typical delay between recruitment of a new neutrophil and that neutrophil becoming apoptotic. Neutrophils have widely been observed to have considerably shorter lifespans than macrophages, broadly in the range of hours to few days [3, 4, 9, 40, 41]. Once again, further details are given in the parameterisation section below.

#### Neutrophil motion

Neutrophils are modelled to move chemotactically toward high concentrations of the pro-inflammatory mediator, *c*. We describe the implementation of this motion in a subsequent section.

#### Release of pro-inflammatory mediators

Neutrophils provide a positive feedback to the inflammatory system, through continuous release of pro-inflammatory mediators [42]. At each tick, each neutrophil acts to increase the pro-inflammatory mediator concentration, *c*, at its current location by an increment *δ*_*nc*_ with probability *p*_*nc*_.

#### Apoptosis

Upon depletion of a neutrophil agent’s lifespan, that neutrophil dies naturally through apoptosis. The biological implications of a neutrophil’s apoptosis in the inflammatory context are multiple and directly affect the complex chain of cellular and chemical interactions described above. In our model, a neutrophil’s apoptosis involves removal of the agent itself (with probability one), and replacing that agent by a new agent of the Apoptotic class, described below.

### Apoptotic neutrophils

**Fig 5:**
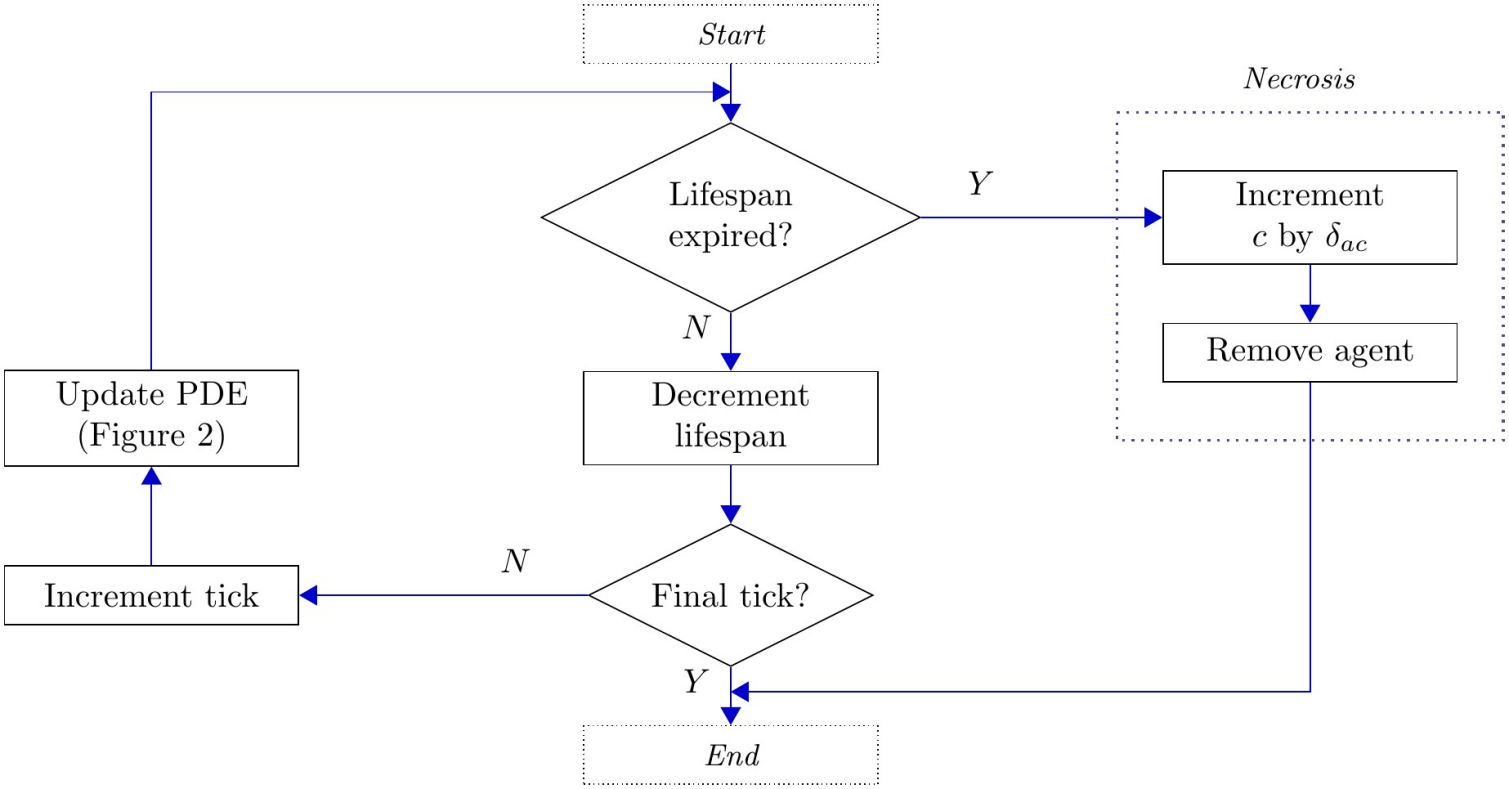
Flow chart illustrating the actions of each agent of the apoptotic class.

Apoptotic neutrophils are considered to be essentially dormant until their eventual necrosis, which we model to occur after a delay drawn from an appropriate distribution, as described in the parameterisation section below. Apoptosis is associated with a significant reduction of cell behaviours, including vastly reduced motility [43]. In our model, we assume that apoptotic cells do not move at all. At the point of its necrosis, an apoptotic neutrophil will release its toxic contents [37, 44, 45], thus generating further tissue damage and an increase in the inflammatory response that here we equate to an increase in pro-inflammatory mediators. Since we do not model tissue damage explicitly here, we incorporate the effects of necrosis of apoptotic neutrophils as a direct source of pro-inflammatory mediators, which we assume to occur with probability one on the necrosis of each apoptotic neutrophil. When an apoptotic neutrophil undergoes necrosis, the pro-inflammatory mediator concentration, *c*, at the cell’s current location is increased by a quantity *δ*_*ac*_. That agent is then immediately removed from the system. Fig 5 summarises the limited activity of apoptotic neutrophils at each tick.

### Modelling chemotaxis

We here describe the manner in which we implement chemotaxis of cells toward areas of high concentrations of pro-inflammatory mediators. We implement the same description of chemotaxis for both macrophages and active neutrophils, and in this section use the term ‘cell’ to refer to either of these (but not apoptotic neutrophils, which do not move at all).

It is well documented in previous literature that cells have the ability to integrate multiple external signals that inform their direction of motion, but also exhibit a degree of directional persistence [46, 47]. We, here, construct a model of chemotaxis that takes into account both of these factors, modelling chemotaxis as a biased persistent random walk. We will observe, below, that incorporating persistence in the cells’ motion is key in replicating experimentally-observed cell trajectories. Moreover, many inflammatory conditions are known to involve defective neutrophil chemotaxis [7–9], which we here consider as a transition to weaker sensitivities to external signals and, potentially, a greater degree of persistence of cell motion.

At every tick of our model, each cell moves by selecting a target grid space from the eight options within its Moore neighbourhood. This selection is governed by some probabilistic rule, which we construct in function of both information on the gradient of *c* in that neighbourhood and the cell’s ‘memory’ of its most recent direction of movement. That is, we construct a probability distribution of the form

$$\begin{array}{c} p=p^{grad}\cdot p^{mem}, \end{array}$$ (3)

in which *p*^*grad*^ describes the probability of moving in each direction in response to the chemoattractant gradient in the absence of cell memory effects, and *p*^*mem*^ describes the probability of moving in each direction in the absence of any chemoattractant, based purely on the cell’s persistence of motion.

Let us consider a generic cell *i*, with neighbourhood $N_{i}$. For each position $j\in N_{i}$, the probability that the cell moves to position *j* (in the absence of memory effects) is a function of *c*_*j*_ − *c*_*i*_ (*i.e.* the difference in chemoattractant concentrations between position *j* and the current position). We prescribe

$$\begin{array}{c} p_{j}^{grad}=\frac{w(c_{j}-c_{i})}{\sum_{k\in N_{i}}w\left(c_{k}-c_{i}\right)}, \end{array}$$ (4)

where *w*(*x*) is a potentially nonlinear weighting function that controls the strength of the dependence upon the chemoattractive signal, and the summation on the denominator is simply a normalisation that ensures that these probabilities sum to unity. Here, we choose

$$\begin{array}{c} w\left(x\right)=\exp\left(k_{grad}x\right), \end{array}$$ (5)

where *k*_*grad*_ is a scaling parameter, which we will later infer from experimental data.

In order to incorporate cell persistence, cellular agents are embedded with a record of the direction of their previous move, *θ*_*prev*_. If *θ*_*prev*_ = 0 for a given cell, that cell’s most recent move was to the right; if *θ*_*prev*_ = *π*/2, the cell’s most recent move was upwards, *etc*. The probability associated with the cell’s memory (*p*_*mem*_) follows a Gaussian distribution, in which the most likely next move is that associated with the preservation of direction. The possibilities for the cell’s next move correspond to the angles *θ*_*j*_ = (*j*−1)*π*/4, for *j* = 1, …, 8. The probability that the cell proceeds by moving in a direction prescribed by the angle *θ*_*j*_ (given *θ*_*prev*_) is given by

$$\begin{array}{c} p_{j}^{mem}=\frac{1}{K}(\frac{1}{\sqrt{2\pi \sigma_{mem}^{2}}}e^{-\frac{{\left(\theta_{j}-\theta_{prev}\right)}^{2}}{2\sigma_{mem}^{2}}})\;, \end{array}$$ (6)

where *K* is a normalising constant that accounts for the fact that the Gaussian is sampled at only eight discrete points, and ensures probabilities sum to unity. The extent to which a cell is likely to change direction is regulated via the standard deviation parameter *σ*_*mem*_, which we once again infer from experimental data below.

Finally, we combine the two facets of cell motion above, and prescribe the probability that cell *i* moves to position $j\in N_{i}$ according to

$$\begin{array}{c} p_{j}=\frac{p_{j}^{grad}\cdot p_{j}^{mem}}{\sum_{k\in N_{i}}(p_{k}^{grad}\cdot p_{k}^{mem})}, \end{array}$$ (7)

where, once again, the denominator provides suitable normalisation. We investigate the sensitivity of our model to choices of the parameters *k*_*grad*_ and *σ*_*mem*_, in particular, in subsequent sections.

### Parameterisation

We here consider, initially, the spatial and temporal scales associated with the ABM described above, paying particular attention to the quantities appearing in the mediator equations of (1) and (2). Within our model, it is natural to consider the resolution of the grid to be such that each grid space corresponds to one cell diameter. We choose to set each grid space to correspond to the diameter of a macrophage, which is approximately 20 *μ*m. For a 100 × 100 grid, this corresponds to a square domain of width 2 mm. When considering the nondimensionalisation required to arrive at the dimensionless PDEs (1) and (2) from dimensional analogues, it is computationally convenient to scale lengths against the size of one grid space (since this provides a mesh with unit spacing that can be coupled easily to the agent-based portion of the model). We therefore deploy a spatial scaling L* = 20 *μ*m (with stars denoting dimensional parameters here) and run simulations on the domain [0, 100] × [0, 100]. Similarly, we are required to choose an appropriate scaling of time, T*. In doing so, we configure the cell velocities against data reported in the literature. Macrophage velocities are reported to lie in the approximate range 0.5–30 *μ*m/min [48–50]. We therefore set each tick to be equivalent to 1 minute (*i.e.* T* = 1 min) and allow macrophages to move once per tick, resulting in a typical macrophage velocity of 20 *μ*m/min. Meanwhile, neutrophils are reported to move roughly twice as fast as macrophages, with average velocities in the range 3.5–63.5 *μ*m/min [10, 51, 52]. We therefore allow neutrophils to move twice per tick with a corresponding neutrophil velocity of 40 *μ*m/min. We note, here, that we impose a direct equivalence between one unit of time in the PDEs of (1) and (2) and one tick of our ABM.

Typical (dimensional) mediator diffusion rates are reported to lie broadly in the range 10^−8^ − 10^−6^ cm^2^/sec. (See [53–55] and references therein.) Here, we take dimensional values $D_{c}^{*}≃D_{g}^{*}≃10^{-7}{cm}^{2}/sec\equiv D^{*}$ as our baseline choice, and apply the nondimensionalisation described above to obtain

$$\begin{array}{c} D_{c}=D_{g}=\frac{D^{*}T^{*}}{L^{*2}}=1.5. \end{array}$$ (8)

We take this as our standard baseline value for *D*_*c*_ and *D*_*g*_ in the simulations below. Similarly, the decay rate parameter *γ*_*c*_ in (1) is related to its dimensional equivalent according to $\gamma_{c}=\gamma_{c}^{*}T^{*}$. Following [16], we take $\gamma_{c}^{*}≃3\;{day}^{-1}$, which provides *γ*_*c*_ ≃ 0.002. Again, we take this as our baseline value for *γ*_*c*_ in simulations below, and for ease we also assume *γ*_*g*_ = *γ*_*c*_.

It then remains to consider the scaling of mediator concentrations themselves. It will be of interest below to make comparison with the related PDE–based model of the inflammatory response presented in [17] below, in which pro-inflammatory mediator concentrations were scaled against the production of pro-inflammatory mediator by apoptotic neutrophils on necrosis. Whilst it is difficult to recover the scaling of [17] exactly, we can make a qualitative comparison by setting the amount of pro-inflammatory mediator released by an apoptotic neutrophil (*δ*_*ac*_) equal to one in our model, and accordingly consider mediators to operate on the scale [0, 1]. We therefore choose our initial conditions to include a central circular area of radius *r* within which we perturb the healthy steady state by setting *c* = *c*_0_ (with *c*_0_ ∈ [0, 1]), surrounded by healthy tissue in which *c* = 0. For ease, we assume that there is no anti-inflammatory mediator initially, so that *g* = 0 everywhere in the domain.

In order to infer a biologically realistic proportionality between the maximum number of macrophages and neutrophils within the tissue, we refer to standard measurements of human differential white blood cells. In particular, neutrophils account for 40% to 80% of the total leukocyte population while macrophages have considerably lower proportions, with typical values between 2% − 10% [56]. As such, by recalling that gridpoints are scaled with respect to the diameter of macrophages (∼20 *μ*m) and that we operate over a 2 mm × 2mm domain, we fix the maximum number of macrophages at *m*_*max*_ = 1000 and a limit number of neutrophils four times larger at *n*_*max*_ = 4000.

Finally, we consider appropriate choices for leukocyte lifespans. As discussed above, the amount of time that macrophages and neutrophils remain active in a tissue can vary significantly between different tissues and in different inflammatory contexts. Macrophages can typically be active from several days to months [30, 31], while the lifespan of neutrophils is generally considerably shorter, from within hours to a few days [3, 4, 9, 40, 41]. Thus, recalling that according to our parameterisation one tick is equivalent to one minute, we model this information by randomly selecting macrophage lifespans from a uniform distribution on the interval [1440, 86400] (*i.e.* 1–60 days) and neutrophil lifespans from a uniform distribution on the interval [60, 1440] (*i.e.* 1–24 hours). For apoptotic neutrophils, there is a lack of experimental methods to properly detect and measure the necrosis timescales [57], but it is generally understood that this a rapid process [58]. We thus prescribe an approximate lifespan for apoptotic neutrophils randomly assigned from a uniform distribution on the interval [60, 720] (*i.e.* 1–12 hours).

Many of the remaining parameters in our model are not known exactly, but some can be inferred qualitatively from biological intuition. For example, we expect the production of pro-inflammatory mediators by active neutrophils to be on a scale much smaller than that by apoptotic neutrophils, so *δ*_*nc*_ ≪ *δ*_*ac*_. Similarly, due to the scaling of mediator concentrations, we intuitively expect *δ*_*mg*_, *α*_*ncr*_, *α*_*ngr*_, *α*_*mr*_, *α*_*ml*_ ∈ [0, 1]. For those parameters whose values are not directly available from existing literature, we choose baseline values that best display the model’s scope for switching between chronic and healthy outcomes. A summary of our model parameters, together with baseline values used in the simulations below, is given in Table 1. We calibrate the parameters associated with cell chemotaxis by comparison with the existing experimental data of [10] below, and also investigate the sensitivity of the model to variations in key parameters in subsequent sections.

**Table 1:** Model parameters, together with baseline values used in the simulations below. PDE parameters are inferred from existing literature as described in the text. Chemotaxis parameters are computed via comparison with the experimental data of [10]. The remaining parameters are mostly unavailable in existing literature, and are thus estimated based on simulation. The model’s sensitivity to these parameter choices is investigated in more detail in the Results section below.

Parameter	Definition	Value
**Initial Conditions**		
*c*_0_	severity of perturbation to the healthy state	1
*r*	radius of perturbed area	10
**Mediator (PDE) Parameters**		
*D*_*c*_, *D*_*g*_	diffusion coefficients	1.5
*γ*_*c*_, *γ*_*g*_	decay rates	0.002
**Source Terms**		
*δ*_*ac*_	mediator *c* released by apoptotic neutrophils	1
*δ*_*nc*_	mediator *c* released by active neutrophils	0.001
*δ*_*mg*_	mediator *g* released by macrophages	0.001
**Probabilities**		
*p*_*nr*_	neutrophil recruitment	0.02
*p*_*nc*_	release of mediator *c* by active neutrophils	0.5
*p*_*mr*_	macrophage recruitment	0.04
*p*_*mg*_	release of mediator *g* by macrophages	0.8
*p*_*ml*_	macrophages leaving the tissue	0.8
*p*_*ma*_	macrophage phagocytosis of apoptotic neutrophils	1
**Thresholds**		
*α*_*ncr*_	minimum mediator *c* for neutrophil recruitment	0.05
*α*_*ngr*_	maximum mediator *g* for neutrophil recruitment	0.015
*α*_*mr*_	minimum mediator *c* for macrophage recruitment	0.4
*α*_*ml*_	minimum mediator *c* for macrophages to remain	0.02
**Chemotaxis Parameters**		
*k*_*grad*_	strength of response to chemoattractant—healthy regime	80
	—impaired regime	8
*σ*_*mem*_	cell persistence—healthy regime	1
	—impaired regime	1.2
**Miscellaneous**		
*n*_*max*_	maximum number of neutrophils	4000
*m*_*max*_	maximum number of macrophages	1000

## Results

In this section, we begin by examining a reduced model in which we simulate the motion of a population of neutrophils toward a fixed chemoattractive target. In doing so, we compare our simulations with existing experimental results in order to inform our choices of the chemotactic parameters *k*_*grad*_ and *σ*_*mem*_. In this reduced model, we neglect macrophages, apoptosis of neutrophils and all inflammatory interactions, and study cell motion alone. Having identified suitable choices of the motily parameters, we deploy these in simulations of our full model, before investigating the model’s sensitivity to variations in the remaining parameter values.

### Calibrating the chemotaxis model

Defects in neutrophil recruitment result in a delayed progression of inflammation, potentially preventing the restoration of a healthy state and leading the way to diseases [59]. Understanding the conditions that prevent the correct migration of leukocytes to the sources of damage upon an inflammatory event is key to developing effective therapeutic tools, in order to contain and eventually tackle such disorders.

The study of Sapey *et al.* [10] investigated the differences in neutrophil chemotaxis between healthy controls and patients affected with chronic obstructive pulmonary disease (COPD)—a disease associated with airway inflammation, increased neutrophil recruitment, and aberrant neutrophil migratory dynamics. In their study, Sapey *et al.* use time-lapse microscopy to monitor the spatial trajectories of populations of neutrophils as they migrate in the presence of a linear gradient of the chemoattractant interleukin–8 (IL–8). In doing so, they record average cell speeds, cell velocities in the direction of the attractant, and a mean chemotactic index, which represents the extent to which cell trajectories align with the direction of the chemotactic gradient. We compare our simulations directly against the results from [10] in order to infer appropriate choices of our chemotaxis parameters *k*_*grad*_ and *σ*_*mem*_, in both healthy and inflamed situations.

In order to compare our simulations with these experiments, we introduce a nondimensionalisation (used in this section only) that compares directly with the experimental setup. In particular, we choose to set each grid space to correspond to 1 *μ*m and, given that the time-lapse images of [10] represent an area of approximately 240 *μ*m × 180 *μ*m, we consider a 240 × 180 rectangular grid as our domain. We consider a distribution of chemoattractant with dimensionless concentrations of zero on the lower boundary and one on the upper boundary, with no lateral variation. Here, we run simulations corresponding to the 20 min duration of experiments presented. Given that the only temporally-dependent behaviour in this reduced model is cell motion itself, the precise definition of one tick in our simulations is arbitrary, given that we have the option to permit multiple cell moves per tick. Given observed cell velocities from [10], and for fixed choices of *k*_*grad*_ and *σ*_*mem*_, we compute the expected number of moves needed to travel the required vertical distance in 20 min. Having done so, we also compute the mean chemotactic index associated with an expected trajectory. The resulting pairs of *k*_*grad*_ and *σ*_*mem*_ values that yield the chemotactic indices measured in [10] are carried forward as viable parameter values for subsequent simulations.

Let us describe a generic cell’s trajectory in terms of a series of individual moves, denoting by *m*_*k*_ the cell’s *k*^*th*^ move. Each cell move is one of the eight options illustrated in Fig 1, which we number from one to eight; if *m*_*k*_ = 1, the cell’s *k*^*th*^ move is to the right; if *m*_*k*_ = 5, the cell’s *k*^*th*^ move is to the left, *etc*. We are thus interested in the probability of the cell selecting move *m*_*k*_, from the eight possibilities, given its previous move *m*_*k*−1_. We define a transition matrix, **P**, such that, at move *k*, *p*_*ij*_ represents the probability that the *k*^*th*^ move is move *j* given that the (*k* − 1)^*th*^ move was move *i*; *i.e.*

$$\begin{array}{c} p_{ij}=P\;\left(m_{k}=j\;|\;m_{k-1}=i\right)\;,\;i,j=1,\ldots ,8\;. \end{array}$$ (9)

At the start of our simulations, agents are initialised with a randomly assigned orientation, each with equal probability, *i.e.*

$$\begin{array}{c} \Pi_{0}=(\begin{array}{cccccccc} \frac{1}{8} & \frac{1}{8} & \frac{1}{8} & \frac{1}{8} & \frac{1}{8} & \frac{1}{8} & \frac{1}{8} & \frac{1}{8} \end{array})\;. \end{array}$$ (10)

The cell’s trajectory is then described by a Markov chain, with transition probability **P** and initial data **Π**_0_, in which each state is one of the eight moves described above. Given this, the probability that the *k*^*th*^ move is move *i* is given by

$$\begin{array}{c} P\left(m_{k}=i\right)={\left(\Pi_{0}\cdot P^{k}\right)}_{i}\;,\;i=1,\ldots ,8\;. \end{array}$$ (11)

Let us now define a new matrix **Q** such that

$$\begin{array}{c} q_{ik}=P\left(m_{k}=i\right)\;, \end{array}$$ (12)

with the right-hand side of (12) being computed according to (11). Given this matrix, we can calculate the expected distance travelled in the vertical direction after *N* moves as

$$\begin{array}{c} d_{v}=\sum_{k=1}^{N}(\sum_{i=2}^{4}q_{ik}-\sum_{i=6}^{8}q_{ik})\;. \end{array}$$ (13)

Given a target cell velocity, we can infer an expected distance of travel from the experimental data, and by comparing this with (13), we prescribe the total number of moves to be carried out within a given simulation. This effectively prescribes the number of moves per tick in our full model, holding fixed the required number of ticks.

Similarly, for each possible move *i*, let us construct a vector ***α*** such that *α*_*i*_ is the angle that the trajectory of move *i* makes with the vertical, *i.e.*

$$\begin{array}{c} \alpha =(\frac{\pi }{2},\frac{\pi }{4},0,\frac{\pi }{4},\frac{\pi }{2},\frac{3\pi }{2},\pi ,\frac{3\pi }{2})\;. \end{array}$$ (14)

We can then compute the expected mean chemotactic index after *N* moves according to

$$\begin{array}{c} I_{chem}=\frac{1}{N}\sum_{k=1}^{N}\sum_{i=1}^{8}\cos\left(\alpha_{i}\right)q_{ik}\;. \end{array}$$ (15)

We use (15) to calibrate our model against the experimental observations of [10] as follows. For a given choice of *k*_*grad*_ and *σ*_*mem*_, and a given cell velocity from [10], we infer the expected vertical distance of travel (*d*_*v*_) over the course of a 20 min experiment, and use (13) to calculate the number of moves (*N*) required to replicate this in our model. Given *N*, we then use (15) to calculate the mean chemotactic index that we expect to observe in our simulations from a theoretical perspective (*I*_*chem*_). We compare this value against the documented mean chemotactic index measurements of [10] to quantify the extent to which our model replicates *in-vitro* cell trajectories for these choices of *k*_*grad*_ and *σ*_*mem*_. We refine our choices of *k*_*grad*_ and *σ*_*mem*_ accordingly, to ensure our simulations reflect *in-vitro* observations, as described below.

**Fig 6:**
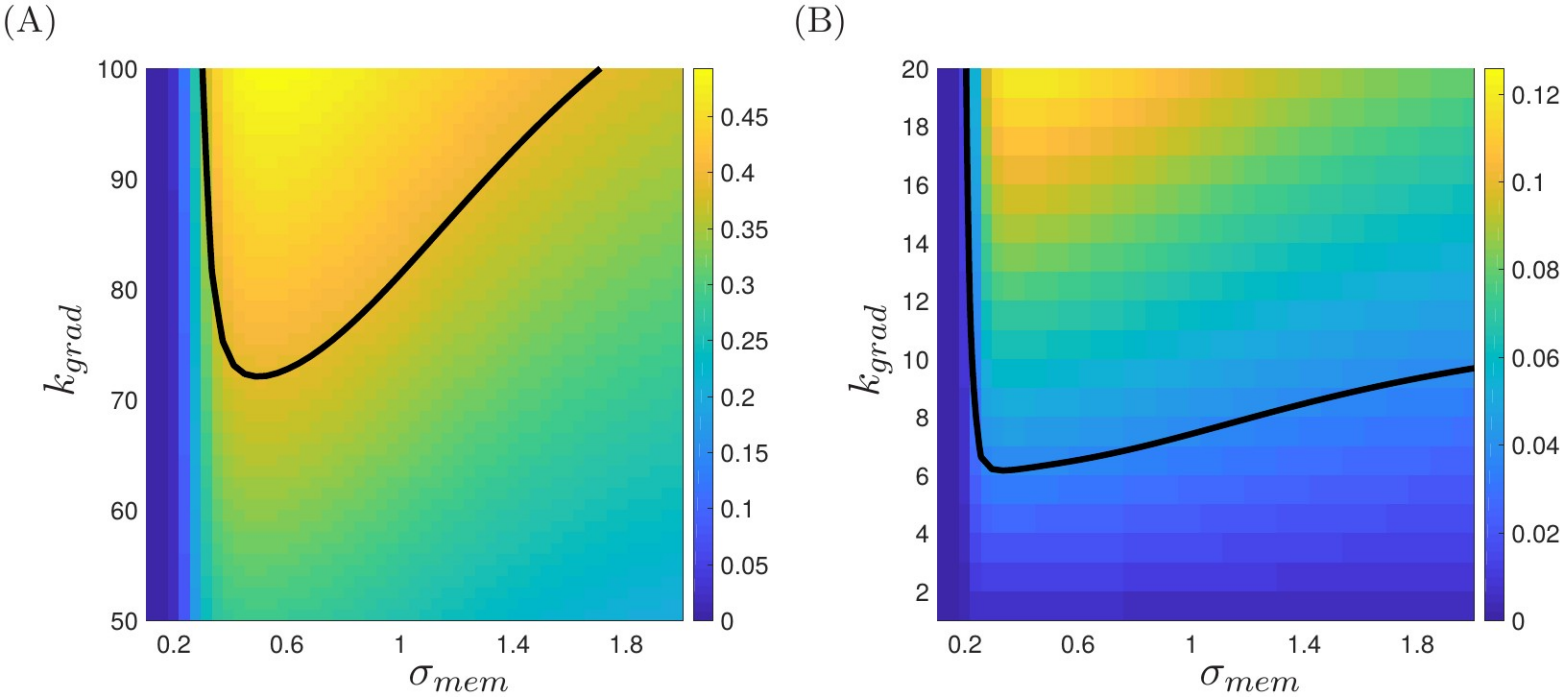
Plots of the mean chemotactic index attained for each combination of *k*_*grad*_ and *σ*_*mem*_. In (A), we calibrate our model against the healthy control data of [10] using a mean cell velocity of 3.77 *μ*m/min; the black line represents a mean chemotactic index of 0.39. In (B), we calibrate our model against the COPD-affected data of [10] using a mean cell velocity of 0.09 *μ*m/min; the black line represents a mean chemotactic index of 0.04.

**Fig 7:**
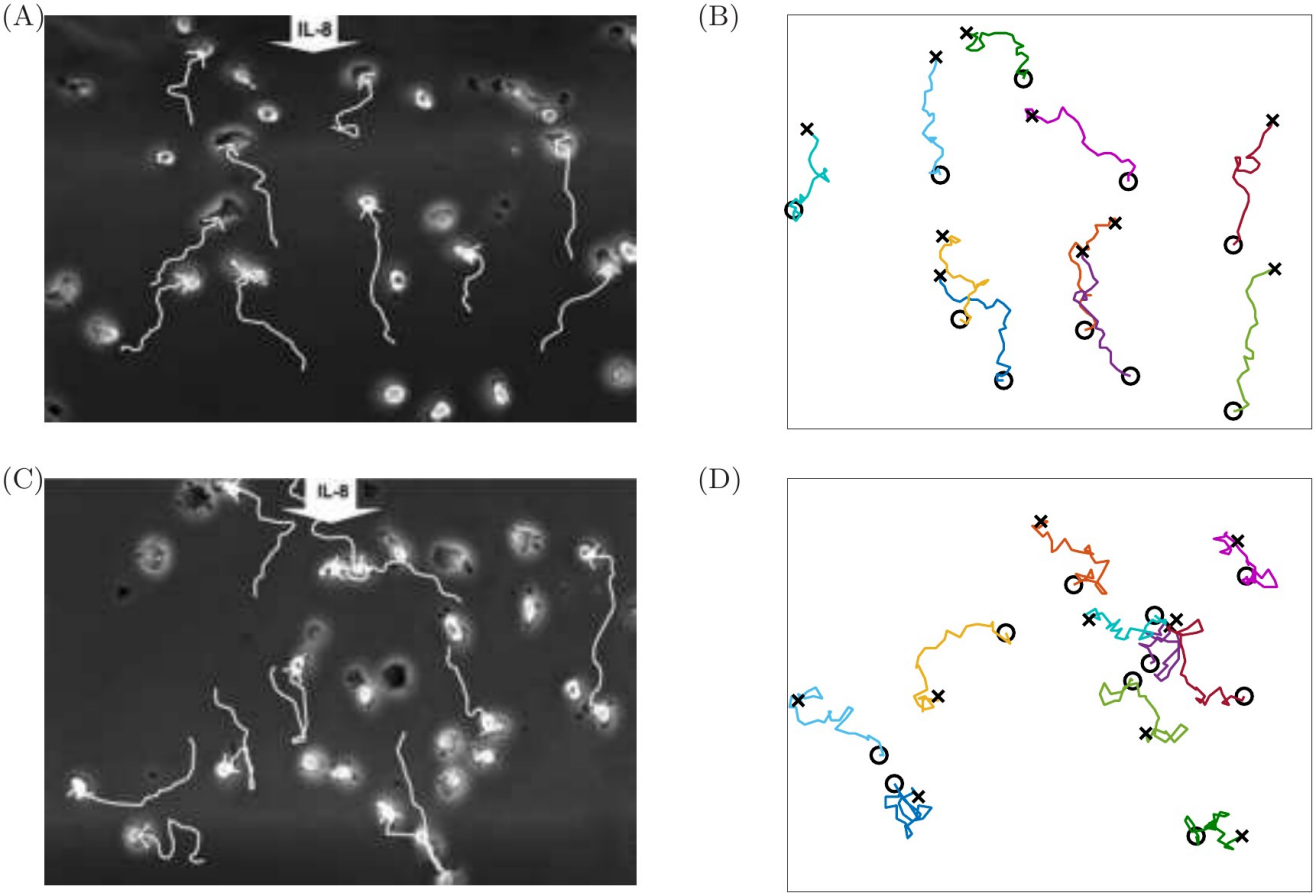
Comparison of the *in vitro* cell tracking experiments of [10] (A,C) with simulations of our calibrated chemotaxis model (B,D). In (A,B), we illustrate the healthy case, with simulations calibrated using a mean cell velocity of 3.77 *μ*m/min and a mean chemotactic index is 0.39, providing *k*_*grad*_ = 80 and *σ*_*mem*_ = 1. In (C,D), we illustrate the inflamed (COPD) case, with simulations calibrated using a mean cell velocity of 0.09 *μ*m/min and a mean chemotactic index is 0.04, providing *k*_*grad*_ = 8 and *σ*_*mem*_ = 1.2. (A,C) Reprinted with permission of the American Thoracic Society. Copyright ©2019 American Thoracic Society. Cite: Sapey *et al.* (2011) ‘Behavioral and structural differences in migrating peripheral neutrophils from patients with chronic obstructive pulmonary disease’, American Journal of Respiratory and Critical Care Medicine 183(9), 1176-1186. The American Journal of Respiratory and Critical Care Medicine is an official journal of the American Thoracic Society.

Fig 6 illustrates the expected mean chemotactic index calculated via (15) for ranges of *k*_*grad*_ and *σ*_*mem*_. In Fig 6A, we show results calibrated using measurements of neutrophils from healthy controls in the study of [10], for which the reported mean cell velocity is 3.77 *μ*m/min and the mean chemotactic index is 0.39. We therefore choose the number of moves, *N*, by requiring that *d*_*v*_ ≃ 75.4 in (13). The heavy black line in the figure illustrates a curve in (*σ*_*mem*_, *k*_*grad*_)–space for which the target chemotactic index is realised. Any pair of parameters that lie on this curve may be chosen as suitable values; we choose *k*_*grad*_ = 80 and *σ*_*mem*_ = 1 to illustrate the corresponding behaviour below. For these parameters our calculation requires that we perform *N* = 160 moves within our simulation. In Fig 6B, we repeat this calculation using measurements of neutrophils from COPD-affected individuals, for which the mean cell velocity is 0.09 *μ*m/min, and the mean chemotactic index is 0.04. We observe that accurate recovery of experimental observations in the healthy case involves a reasonably significant dependence upon the cell persistence parameter *σ*_*mem*_, while the COPD case exhibits a much weaker depedence on cell memory alongside a lesser ability of cells to sense the chemotactic gradient, corresponding to smaller choices of *k*_*grad*_. Below, we choose *k*_*grad*_ = 8 and *σ*_*mem*_ = 1.2 to illustate behaviours in the chemotactically impaired case. Fig 7 illustrates the cell trajectories observed in [10] alongside simulations of our model using each of the parameter sets identified above. We observe reasonably well-directed motion toward the chemoattractant in the healthy case (Fig 7A and 7B), but much less directed motion in the COPD case (Fig 7C and 7D).

### Simulations of the full inflammatory model

Below, we present simulations of the full inflammatory model in both healthy and impaired chemotactic regimes, and for a range of the remaining model parameters. Throughout, we are primarily interested in how variations in model parameters drive switching between chronic outcomes and full resolution of inflammation. Below, we associate chronic outcomes with a sustained presence of pro-inflammatory mediators or active/apoptotic neutrophils. Simulations for which the numbers of pro-inflammatory mediators and active/apoptotic neutrophils all eventually reach zero are identified as healthy outcomes, with inflammation being fully resolved.

**Fig 8:**
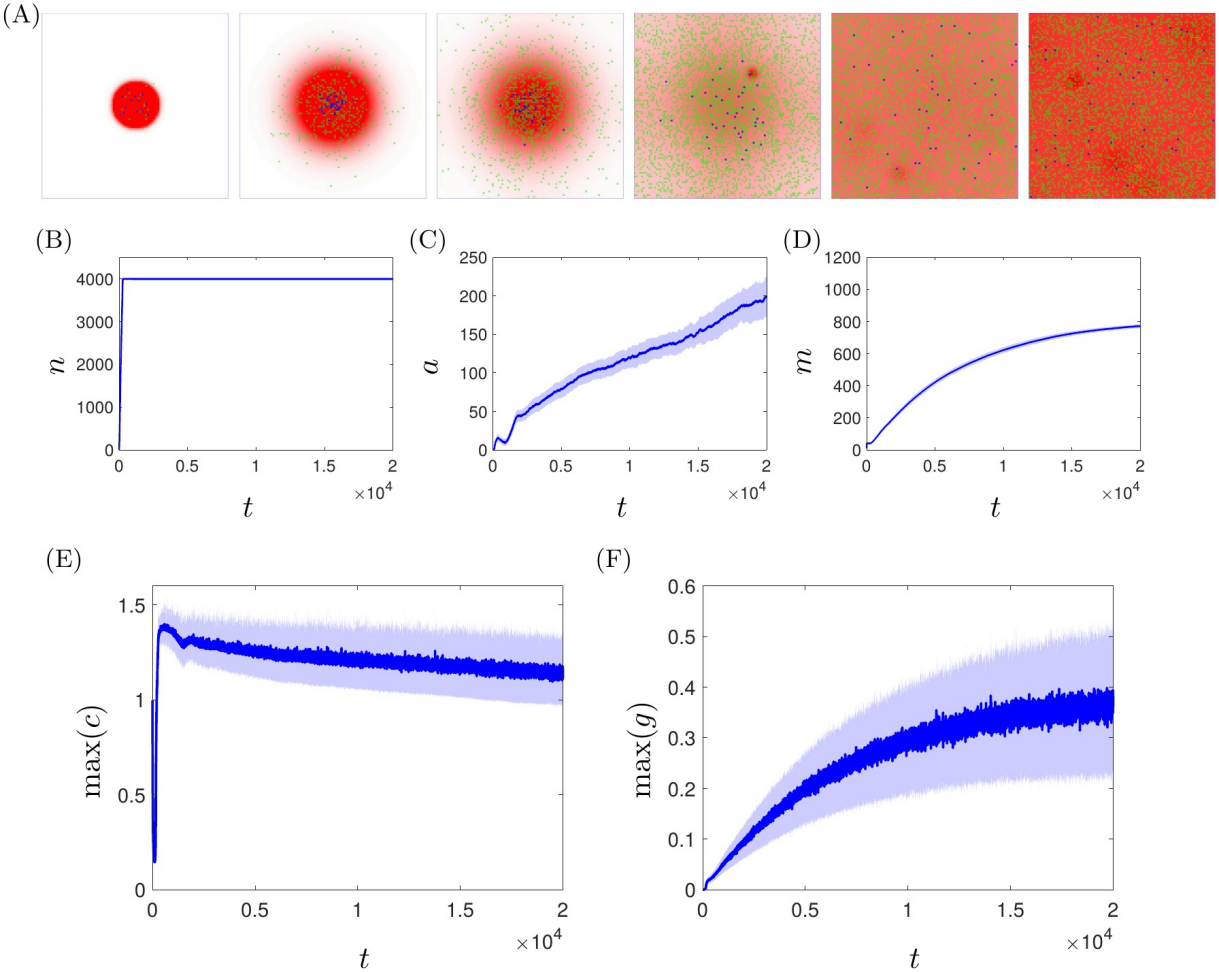
A typical simulation of the inflammation model showing a chronic, globally inflamed outcome, for healthy choices of chemotactic parameters (*k*_*grad*_ = 80, *σ*_*mem*_ = 1) and all other model parameters as given in Table 1. (A) Successive snapshots of the simulation in which high levels of pro-inflammatory mediators are shown in dark reds in the background; active/apoptotic neutrophils are shown in green/orange respectively and macrophages are shown in blue. (B–D) Global cell counts for active neutrophils, apoptotic neutrophils and macrophages respectively. (E,F) Maximal concentrations of mediators *c* and *g* across the domain. Solid lines represent the mean result over 100 simulations of the model; shaded areas represent plus/minus one standard deviation.

In Fig 8 we illustrate simulations of the full inflammatory model, with chemotaxis parameters corresponding to the healthy case described above (*k*_*grad*_ = 80, *σ*_*mem*_ = 1) and the remaining parameter values as given in Table 1. In the figure, we show snapshots of a typical simulation over a range of ticks spanning 0–20,000. Initially, we perturb the healthy steady state via a positive concentration (*c*_0_) of pro-inflammatory mediators in the centre of the domain (which we use as a proxy for tissue damage). This perturbation drives recruitment of both neutrophils and macrophages, with the combined effects of diffusion of pro-inflammatory mediators and the pro-inflammatory feedback from neutrophils ultimately yielding a globally inflamed, chronic state. Given the stochastic nature of our model, we routinely simulate in batches of 100 simulations, and consider the mean response. Fig 8 also shows the mean global cell count for each type of cell (B–D), and the maximal mediator concentrations across the domain (E,F). We observe that the active neutrophil and macrophage populations rapidly grow toward their maximum capacities in response to the initial perturbation, and these levels are sustained due to the sustained presence of pro-inflammatory mediators. Mediator concentrations ultimately reach a pseudo-steady state, with high levels of pro-inflammatory mediators (in particular) sustained indefinitely.

**Fig 9:**
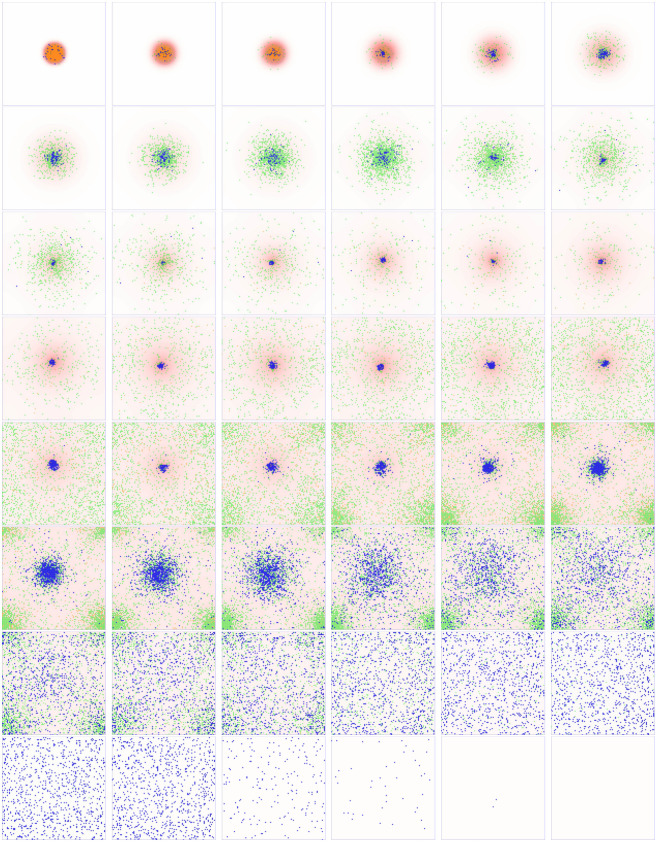
Snapshots of a typical healthy response, for healthy choices of chemotactic parameters (*k*_*grad*_ = 80, *σ*_*mem*_ = 1), *α*_*mr*_ = 0.25 and all other model parameters as given in Table 1. High levels of pro-inflammatory mediators are shown in dark reds in the background; active/apoptotic neutrophils are shown in green/orange respectively and macrophages are shown in blue. Corresponding time-courses showing global cell counts and mediator concentrations are shown in green in Fig 10.

**Fig 10:**
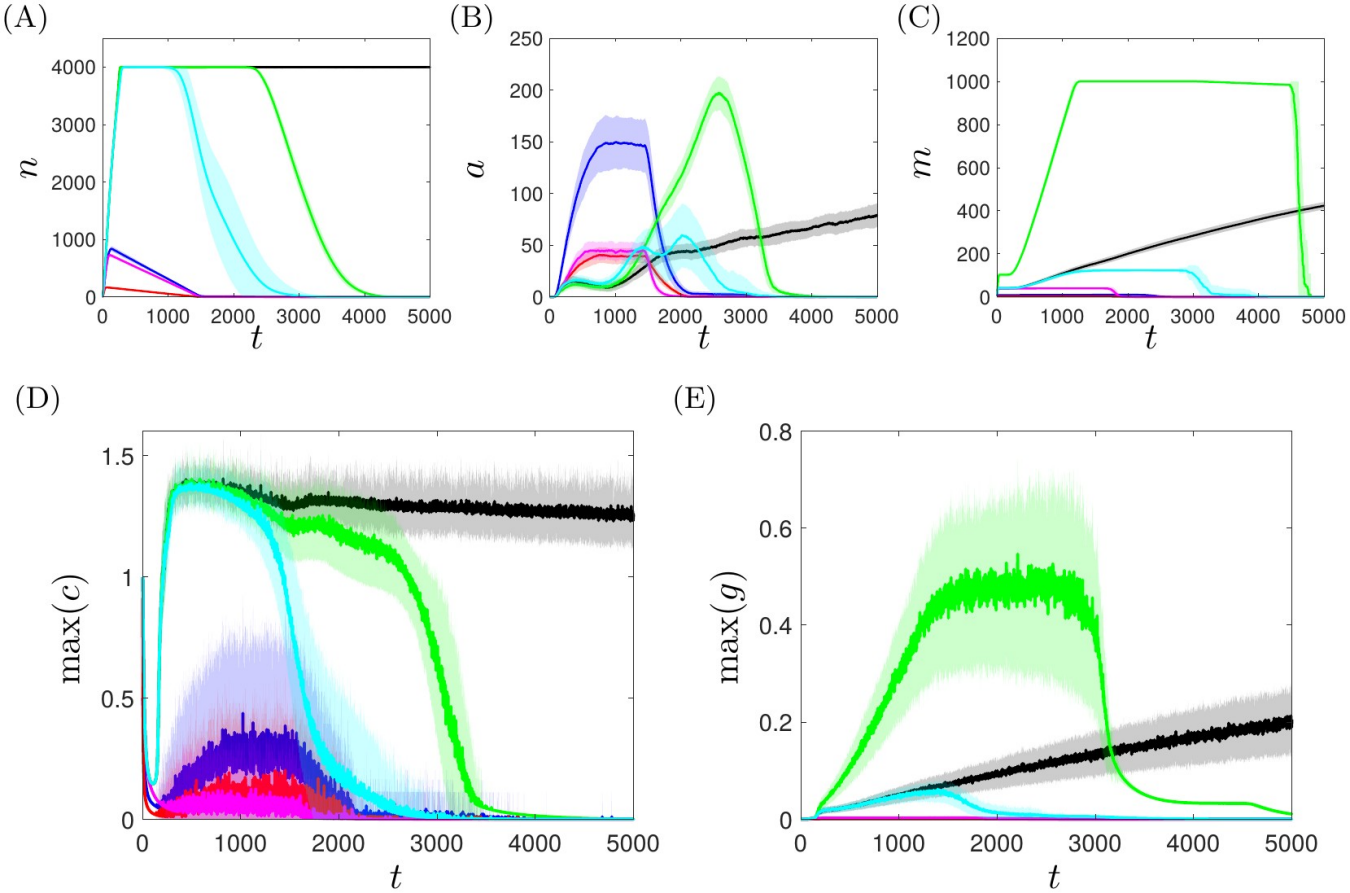
Simulations resulting in healthy, resolved outcomes for *c*_0_ = 0.5 (blue), *r* = 5 (red), *α*_*mr*_ = 0.25 (green), *α*_*ncr*_ = 0.1 (magenta) and *α*_*ngr*_ = 0.0015 (cyan), with healthy choices of chemotaxis parameters (*k*_*grad*_ = 80, *σ*_*mem*_ = 1) and all other parameters as in Table 1. The chronic inflammation of Fig 8 is shown in black. (A–C) Global cell counts for active neutrophils, apoptotic neutrophils and macrophages respectively. (D,E) Maximal concentrations of mediators *c* and *g* across the domain. Solid lines represent the mean result over 100 simulations of the model; shaded areas represent plus/minus one standard deviation.

Given the chronic response yielded by the baseline parameter set of Table 1 (for healthy choices of chemotactic parameters), we observe that manipulation of some key parameters can switch the system to a healthy long-term outcome, in which all of the pro-inflammatory components of the model (*i.e.* *c* and the sizes of the active/apoptotic neutrophil populations) go to zero. Fig 9 illustrates temporal snapshots of a typical resolving simulation. Our simulations reveal that the size and severity of the initial perturbation from the healthy steady state (*r* and *c*_0_ respectively) and the mediator thresholds governing leukocyte recruitment (*α*_*mr*_, *α*_*ncr*_ and *α*_*ngr*_) exhibit the most significant switching behaviour in this regard. As Fig 10 shows, reducing the severity of the initial perturbation by setting either *r* = 5 or *c*_0_ = 0.5 yields a healthy outcome. Similarly, reducing the number of neutrophils recruited by either increasing *α*_*ncr*_ or decreasing *α*_*ngr*_ can yield a healthy response (as shown for *α*_*ncr*_ = 0.1 and *α*_*ngr*_ = 0.0015 in Fig 10). Likewise, we can bias the system toward an eventual healthy state by stimulating a greater anti-inflammatory response through enhanced macrophage recruitment by reducing *α*_*mr*_, as illustrated for *α*_*mr*_ = 0.25 in Fig 10.

**Fig 11:**
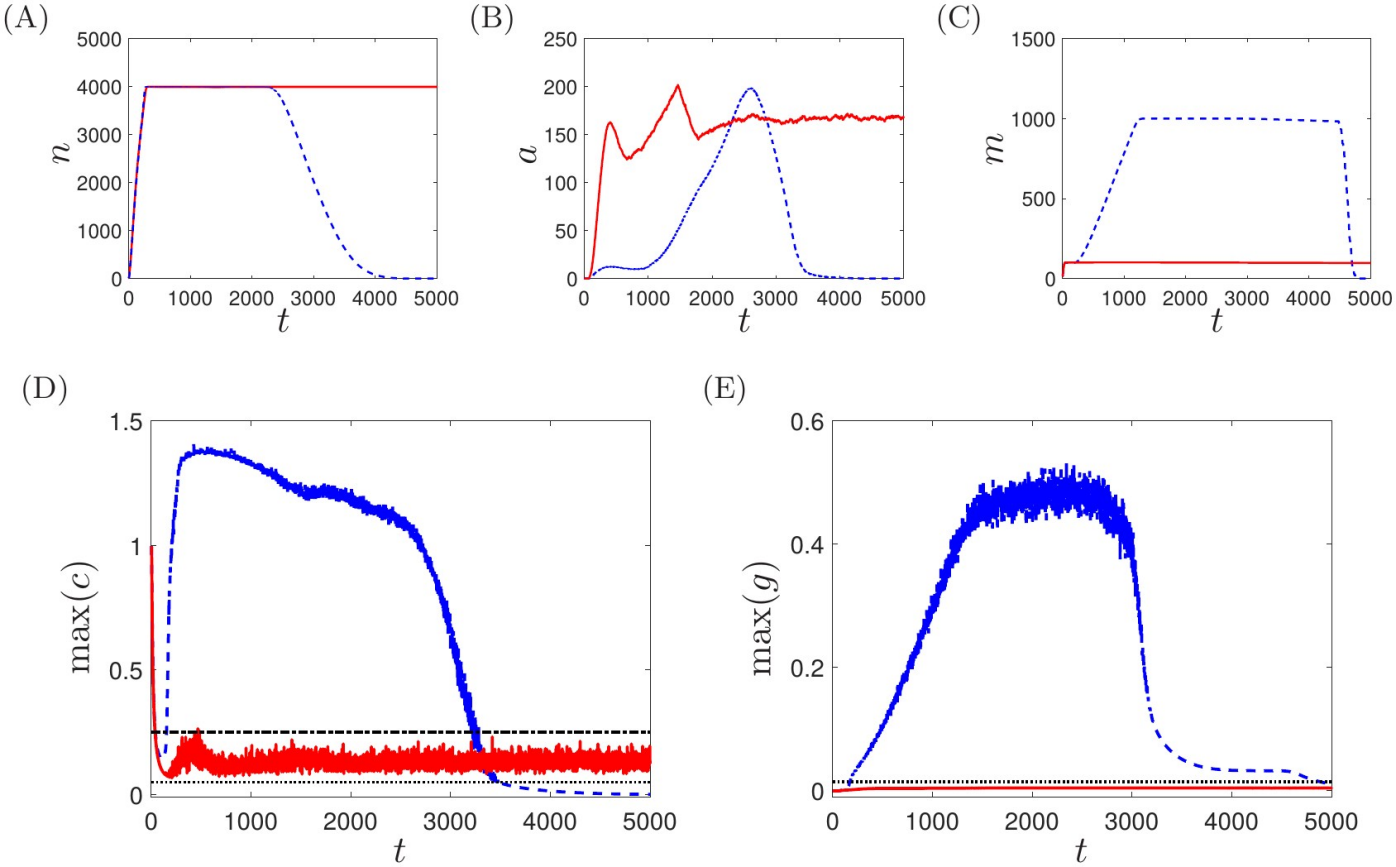
Simulations for *α*_*mr*_ = 0.25 and all other parameters as given in Table 1, with healthy neutrophil chemotaxis (*k*_*grad*_ = 80, *σ*_*mem*_ = 1, shown as dashed blue lines) and impaired neutrophil chemotaxis (*k*_*grad*_ = 8, *σ*_*mem*_ = 1.2, shown as solid red lines). (A–C) Global cell counts for active neutrophils, apoptotic neutrophils and macrophages respectively. (D,E) Maximal concentrations of mediators *c* and *g* across the domain. Dotted lines demark the thresholds for neutrophil recruitment (*c* = *α*_*ncr*_, *g* = *α*_*ngr*_); the dash-dotted line demarks the threshold for macrophage recruitment (*c* = *α*_*mr*_). Impaired neutrophil chemotaxis causes a previously healthy outcome to become chronic.

For parameters yielding a typical healthy response, impaired neutrophil chemotaxis can switch the outcome to one of chronicity, as illustrated in Fig 11, which compares simulations in each chemotaxis regime for *α*_*mr*_ = 0.25 and all other parameters as given in Table 1. For healthy neutrophil chemotaxis (*k*_*grad*_ = 80, *σ*_*mem*_ = 1), neutrophils remain largely localised at the area of the initial perturbation in the short term, resulting in a significant surge in pro-inflammatory mediators in this region that elicits a strong macrophage response. These macrophages then begin to mitigate against further damage by releasing anti-inflammatory mediators and phagocytosing apoptotic neutrophils, resulting in the ultimate restoration of the healthy state. When neutrophil chemotaxis is impaired, neutrophils rapidly migrate away from the site of the initial perturbation. As they do so, they continue to release pro-inflammatory mediators; however, the wider spatial distribution of neutrophils results in these mediators being more spread out across the domain, with local levels falling below the threshold for macrophage recruitment (as shown in Fig 11D). As a result, macrophage populations are much smaller in general, and are not able to generate sufficient anti-inflammatory feedback to allow the inflammation to resolve. The system attains a chronic state, with a low level of pro-inflammatory mediators sustained indefinitely through the continued presence of active and apoptotic neutrophils.

### Parameter sensitivity analysis

**Fig 12:**
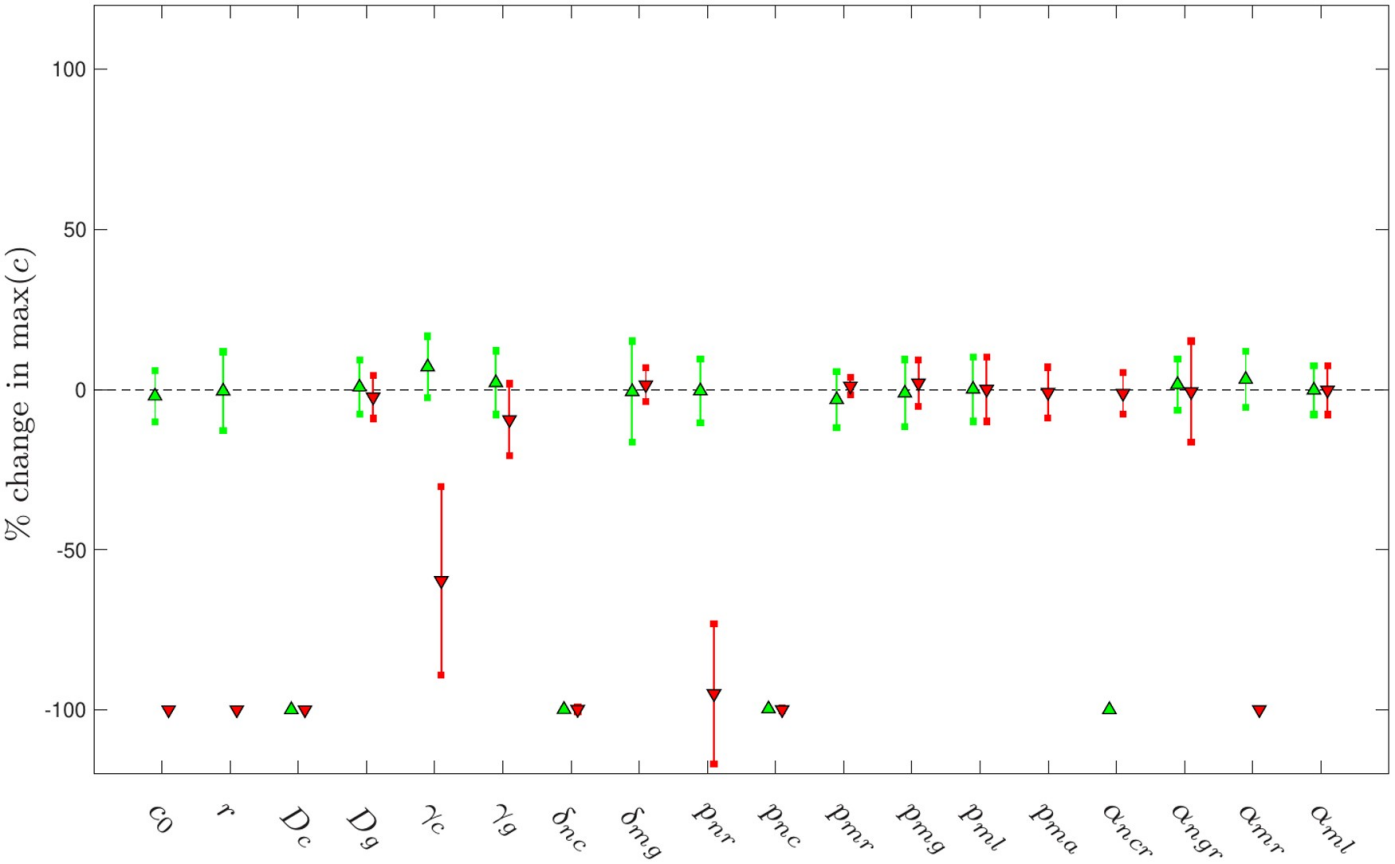
Percentage change in the maximal level of pro-inflammatory mediator *c* at *t* = 5000 on varying each individual parameter by + 50% (green) and −50% (red), from the baseline parameter values given in Table 1, with healthy choices of chemotaxis parameters (*k*_*grad*_ = 80, *σ*_*mem*_ = 1). Triangles denote the mean percentage change in the response; error bars denote plus/minus one standard deviation. (For probabilities for which an increase of 50% results in a choice greater than one, the green results correspond to simulations with unit probability.) Note that a change in response of −100% corresponds to a switch from a chronic to a healthy outcome.

In Fig 12, we examine the extent to which the inflammatory outcome depends upon our choices of model parameters. Holding chemotactic parameters fixed at the healthy choices resulting from the calibration above (*k*_*grad*_ = 80, *σ*_*mem*_ = 1), we perform simulations with each parameter in Table 1 increased by 50% (denoted by upward pointing green triangles in the figure) and decreased by 50% (denoted by downward pointing red triangles in the figure) and record the mean percentage change in the maximal level of pro-inflammatory mediator *c* at *t* = 5000. (We omit the parameter *δ*_*ac*_ from this analysis, as this parameter is directly implicated in the non-dimensionalisation of the model. For parameters representing probabilities for which an increase of 50% would result in a choice greater than one, we instead perform simulations with unit probability.) As usual, all results are averaged across batches of 100 simulations. Since the baseline parameter set of Table 1 yields a chronic outcome (Fig 8), we are particularly interested in whether changes in parameter values can result in a switch to full resolution (*i.e.* a percentage change of −100% in Fig 12).

Intuitively, a reduction in the severity of the initial perturbation (via either *c*_0_ or *r*) can result in the inflammation being fully resolved. Interestingly, the model is highly sensitive to variations in the rate of pro-inflammatory mediator diffusion, *D*_*c*_, in that both high and low choices can drive resolution, by weakening the model’s positive feedback or strengthening its negative feedback respectively. For large choices of *D*_*c*_, pro-inflammatory mediators rapidly spread spatially, attaining low levels across the domain. This results in a much weaker neutrophil response, which is ultimately overcome by macrophages. Conversely, for small choices of *D*_*c*_, pro-inflammatory mediators remain more localised, with greater levels at the site of the initial perturbation. This triggers more rapid recruitment of macrophages, which in turn generate increased release of anti-inflammatory mediators, which once again trigger full resolution. Similarly the model exhibits some dependence upon the pro-inflammatory mediator decay parameter, *γ*_*c*_. For the parameters studied in Fig 12, a reduction of *γ*_*c*_ is sufficient to stimulate a greater macrophage response, yielding a long-term reduction in the levels of pro-inflammatory mediators. The model is much less sensitive to the PDE-parameters associated with anti-inflammatory mediators, *D*_*g*_ and *γ*_*g*_, for the parameters investigated here.

Reducing the recruitment of neutrophils by increasing *α*_*ncr*_ (or decreasing *p*_*nr*_) or stimulating the recruitment of macrophages by reducing *α*_*mr*_ can switch the model to a healthy outcome. The model is less sensitive to choices of the neutrophil recruitment parameter *α*_*ngr*_; however, more significant reductions in this parameter can also drive resolution, as illustrated in Fig 10. The model exhibits a bidirectional sensitivity to the strength of the pro-inflammatory neutrophil feedback (*δ*_*nc*_, *p*_*nc*_) in the same manner as is described for the diffusion parameter *D*_*c*_ above. The model is largely insensitive to variations in the remaining parameters (for the combinations examined here); in particular, while variations in probabilistic parameters related to macrophages may affect the timescales associated with macrophage recruitment and the anti-inflammatory response, these do not affect the long-term inflammatory outcome here. We note that the detrimental effects of reducing the phagocytosis probability *p*_*ma*_ are not readily apparent here, since our parameter sensitivity is conducted with reference to a baseline case that is already chronic; however, simulations conducted for parameter sets that generally yield a healthy response can exhibit a switch to chronicity under a reduction of *p*_*ma*_. These results are omitted for concision. The parameter *α*_*ml*_ does not effect a switch between healthy and chronic outcomes, here.

We remark, here, that the parameter sensitivity analysis conducted here restricts attention to variation of individual parameters in isolation only. Given the high dimensionality of the parameter-space in which this model resides, it is likely that further switching behaviour can result on varying parameters in tandem. While a global parameter sensitivity analysis would be beneficial in further elucidating parameter dependence, the computational cost of performing such an analysis presents a barrier here. While our parameter sensitivity analysis has revealed the roles of various parameters/behaviours in switching between chronicity and restoration of health above, it is perhaps equally pertinent to note that the model seems to exihibit relatively low dependence upon our choices of many of the probabilistic and threshold parameters given in Table 1. Given that these are the parameters whose values we have the least confidence in, Fig 12 provides valuable reassurance that our model conclusions are relatively robust to our choices of these parameters.

## Discussion

We have constructed a hybrid PDE–ABM of the acute inflammatory response, which elucidates the roles of key constituent mechanisms in controlling the switch from chronic inflammation to healthy outcomes (or *vice versa*). Our model illustrates that the long-term inflammatory outcome is determined by two broad categories of behaviours. Firstly, there is a complex and delicate repertoire of spatially-independent interactions that represent the pro-inflammatory (positive) feedbacks of neutrophils and the anti-inflammatory (negative) feedbacks of macrophages. Conditions that enhance the neutrophil response (via *e.g.* *δ*_*ac*_, *δ*_*nc*_ large or *α*_*ncr*_ small in our model) are more likely to yield a self-perpetuating inflammatory condition, while treatments that enhance the macrophage response (via *e.g.* *δ*_*mg*_ large or *α*_*mr*_ small) are more likely to stimulate resolution in the long-term. (See Figs 9 and 12.) Mitigating against inflammation by actively manipulating the synthesis of anti-inflammatory mediators, in particular, remains an active area of research in the ongoing search for new treatments [60–62]; however, we are not currently aware of any clinical interventions that explicitly manipulate macrophage number. Secondly, we have shown that efficient leukocyte chemotaxis is key in controlling the spread of inflammation driven by spreading pro-inflammatory mediators and potentially aberrantly migrating neutrophils, with impaired chemotaxis having the potential to switch a healthy configuration to one of chronicity. While these mechanisms are distinct in some sense, it is key to note that cellular interactions are reliant upon the required components occupying the same area of space, and as such these behaviours are inextricably linked. Our model provides insight into how these mechanisms could potentially be manipulated, either individually or in tandem, in the ongoing hunt for treatments.

Our model pays particularly close attention to how to accurately replicate leukocyte trajectories under healthy and impaired chemotactic regimes. Aberrant neutrophil migration is heavily implicated in myriad inflammatory conditions (as described in, *e.g.*, [8, 63, 64]), and is an attractive target for treatment of various inflammatory conditions, such as COPD [9, 10]. Furthermore, neutrophils from older but otherwise healthy adults are known to be dysfunctional, having a reduced ability to move towards sites of inflammation [8]. This loss of motility in neutrophils has also been found to be worse in infection, such as bacterial pneumonia that often follows viral infection or sepsis [65]. As such, targeting neutrophil motility is now of strong interest as a potential therapeutic target; however, the dual role of neutrophils renders it critical that such interventions are carefully investigated to ensure that necessary phagocytic functions are facilitated while host damage is also prevented [65]. While our leukocyte migration model is deliberately simple in its design, we have shown (via comparison with the spatial data of [10]) that its parameterisation provides sufficient scope to be tuned against experimental data to recover *in-vitro* cellular trajectories well. We note that more complicated models of both individual and collective cell migration that include detailed descriptions of relevant cellular and sub-cellular mechanisms are available in existing literature—see *e.g.* [66–68] and references therein; however, their implementation within an agent-based framework would be largely more cumbersome than the phenomenological approach used here. Our simulations show (in Fig 11) that in an impaired chemotactic regime, in which cells are equipped with a weaker sensitivity to the chemotactic target (*i.e.* reduced *k*_*grad*_) and a greater persistence in their direction of motion (*i.e.* increased *σ*_*mem*_), an otherwise healthy response can be pushed to a self-perpetuating inflamed state (for all other parameters unchanged). This observation is of key importance in two regards: not only do these simulations reveal that impaired leukocyte chemotaxis can act as an independent switch between health and chronicity; they also illuminate the importance of careful calibration of chemotaxis models against experimental data, without which models could easily generate deceiving results.

Previous mathematical models of the inflammatory response have generally adopted a differential-equation-based approach, and generally focus upon relatively simple descriptions of generic populations of leukocytes, potentially omitting some key cellular interactions for mathematical tractability. Examples include the models of [11–14, 69], amongst others. The recent work of Bayani *et al.* [17] complemented these models with a spatially-dependent (PDE) description of the classes of leukocytes and inflammatory mediators described here, to examine the extent to which motility of these components can cause localised damage to invade neighbouring healthy tissue. Many of the observations of the model described herein are consistent with those of [17]. Our model is highly sensitive to changes in the rate of diffusion of pro-inflammatory mediators, *D*_*c*_, with rapid diffusion eliciting only a weak neutrophil response and slow diffusion triggering a strong but localised anti-inflammatory response via macrophages, both of which can restore the healthy state. The model of [17] demonstrated that rapid diffusions in general drive restoration of the globally healthy state, and that efficient neutrophil chemotaxis can act to resolve spatially inhomogeneous inflammatory patterns, which is consistent with our findings above. The models of [16] and [17] also exhibit strong sensitivity to the strength of the (positive) neutrophil feedback, with weak neutrophil feedback yielding a bistable regime in which both healthy and chronic outcomes are stable, and very strong neutrophil feedback resulting in a greater macrophage response that guarantees that a healthy outcome is attained. This is consistent with our observations that the hybrid model presented here exhibits a bidirectional sensitivity to the strength of this feedback via the parameter *δ*_*nc*_ (as shown in Fig 12). Furthermore, the rate of phagocytosis of apoptotic neutrophils has been shown to play a critical role in determining inflammatory outcomes, with the previous models of [16] and [17] showing that impaired phagocytosis results in the only permissible outcome being one of chronicity, while efficient phagocytosis yields bistability with both healthy and chronic configurations possible. This is consistent with the observations of our current model, in which large choices of *p*_*ma*_ provide both chronic and healthy outcomes (in a manner dependent upon other model parameters; Fig 9), but small choices of *p*_*ma*_ eliminate the scope for healthy outcomes.

The hybrid model presented here has facilitated inclusion of a greater array of cell-specific behaviours than is afforded by many previous (mostly ODE- or PDE-based) models. For example, the model includes a description of cells’ chemotaxis toward pro-inflammatory mediators that is calibrated against experimental data, and also specifically incorporates the preferential motion of macrophages toward nearby apoptotic neutrophils (a chemotactic behaviour that was omitted in the model of [17], for example). Furthermore, the model includes an explicit (while simplistic) description of the activation of the anti-inflammatory macrophage response that can be applied on a cell by cell basis as each macrophage undergoes its first phagocytosis of an apoptotic neutrophil. The precise cellular and sub-cellular mechanisms that initiate production of anti-inflammatory mediators are complex (see *e.g.* [6] for details); however, the link between the phagocytosis of apototic cells and the phenotypic switch of macrophages from the classically activated M1 phenotype to the alternatively activated, anti-inflammatory M2 phenotype is well documented in existing literature [70–72]. This notwithstanding, it is well-known that the M1/M2 classification of macrophages presents a degree of over-simplification in itself, with the broad range of macrophage phenotypes actually spanning a continuous spectrum [6]. There is a great degree of scope to extend our model to include more detailed descriptions of macrophage phenotypes and corresponding inflammation-related behaviours going forward. In the model presented here, we include only the anti-inflammatory effects of the M2 macrophage; however, macrophages of the M1 phenotype can also provide pro-inflammatory stimuli, which are here omitted as we focus on the dominant pro-inflammatory effects of neutrophils. Our model also omits the scope for bidirectional switching between macrophage phenotypes. More refined modelling of the relevant cell signalling cascades that govern phenotypic switching of macrophages remains a target for future study.

Our model exhibits significant scope to be calibrated to model specific inflammatory conditions in specific tissues. An open question, in this context, is that of how to infer some parameter values directly from experiments. While the PDE parameters (*D*_*c*_, *D*_*g*_, *γ*_*c*_, *γ*_*g*_) can be inferred from existing literature to some extent, and chemotactic parameters (*k*_*grad*_, *σ*_*mem*_) can be inferred from cell tracking experiments as described here, there remains a degree of uncertainty regarding the various threshold parameters controlling cell recruitment (*α*_*ncr*_, *α*_*ngr*_, *α*_*mr*_) and the strengths of the corresponding inflammatory feedbacks (*δ*_*nc*_, *δ*_*ac*_, *δ*_*mg*_). These parameters are likely to vary across both inflammatory conditions and affected tissues. We have the least confidence in the precise values of the probabilistic parameters in the model; however, we have also shown that in most cases the model’s outputs are robust to variations in these values (Fig 12). Furthermore it is to be expected that our model exhibits some sensitivity to the choice of how cellular responses are scheduled ‘per tick’, or equivalently how a tick itself is defined. We note that tick definition affects only the agent-based components of our model, since numerical solution of the PDEs is implemented independently. In our implementation, most behaviours are scheduled to occur at every tick. We anticipate that alternative implementations of most behaviours simply correspond to alternative choices of the related probabilities or feedback parameters, and thus would not impact upon the qualitative observations presented here. The only behaviour that is not scheduled every tick here is cell recruitment, which we schedule every two ticks for neutrophils and every five ticks for macrophages to account for more rapid recruitment of neutrophils *in vivo*. Simulations that experiment with alternative choices of these recruitment frequencies (omitted here for brevity) reveal that the model is relatively robust to variations of these values. For parameter choices that yield a healthy outcome here (*e.g.* *α*_*mr*_ = 0.25; Figs 9 and 10), alternative choices of cell recruitment frequencies generally still result in a healthy outcome, but with some small variation in the time taken to eliminate pro-inflammatory components fully. For a parameter-set corresponding to a typical chronic simulation (*e.g.* that of Fig 8), small variations in recruitment frequencies make minimal difference to model results; however, significantly slower neutrophil recruitment or significantly more rapid macrophage recruitment can yield a switch from chronicity to restoration of health in some cases. Experimental studies that quantify cell recruitment, in particular, would advance our ability to model accurately specific inflammatory conditions.

We note that, while our model pays close attention to leukocyte movement within the tissue of interest, the description of how leukocytes arrive from the vasculature was kept deliberately simple. In reality, cell transmigration from blood vessels depends on a complex set of events that reduce the velocity of cells flowing in the blood and enable cell adhesion to the endothelial lining, resulting in the endothelial lining itself activating to allow cells to migrate through the vessel wall into the tissue. [73] While including all of these events would be unnecessarily complex in a model of generic inflammation such as that presented here, our model could easily be adapted to include feedbacks from mediators that enhance or restrict transmigration. We note that doing so may involve replacing existing rules governing maximum global cell numbers with more localised analogues that account for spatial variations in endothelial lining activation. Such modifications constitute a valuable target for future work, but would be best addressed when suitable experimental data are available to focus the application of the model toward answering questions related to specific tissues or inflammatory conditions.

There is great scope to develop upon our model further in the future, to study specific tissues, ailments or clinical interventions. Furthermore, there is currently significant and growing interest in multimorbidity, *i.e.* the concurrent presence of two or more chronic conditions such as COPD and cardiovascular disease, which is more common with increasing age and is thought to be associated with inflammation and cellular dysfunction [74]. Understanding how the inflammatory process is modified by disease, healthy ageing and drugs, both alone and in combination, is difficult but necessary if therapeutic targets are to be identified and their effects fully understood. The model presented here is generic but can act as a framework within which future modifications in line with specific tissue and/or disease can be easily incorporated. An example would be modelling the effects of Rheumatoid arthritis on the synovium where macrophage numbers have been shown to correlate with disease activity and their depletion has a therapeutic effect [75, 76]. Such adaptation would require, among others, changes to our description of macrophage heterogeneity but could allow clinical investigations into the effects of drugs that target circulating monocytes and thereby reduce macrophage transmigration into tissue [75]. The advantage of hybrid models, which integrate constituent underlying processes across multiple scales, is that they are easily comparable to experimental data (such as histological studies) and offer easily interpreted tools that could be used in progressing our understanding of such complex, multifaceted inflammatory scenarios.
